# Sustainable Poly(Lactic Acid)/Graphene Oxide Bioelectronic Platform for Neurotransmitters Sensing and Tunable Stimulation of Astrocytes

**DOI:** 10.1002/adma.74487

**Published:** 2026-08-09

**Authors:** Alessandra Scidà, Giulia Cazzador, Roberta Fabbri, Matteo Calosi, Alessandro Silvestri, Andrea Candini, Fabiola Liscio, Franco Corticelli, Aikaterini Konstantoulaki, Andrea D'Iorio, Marco Caprini, Vincenzo Palermo, Monica Bertoldo, Valentina Benfenati, Chiara Zanardi, Emanuele Treossi

**Affiliations:** ^1^ Institute of Organic Synthesis and Photoreactivity (ISOF) – National Research Council of Italy (CNR) Bologna Italy; ^2^ Department of Molecular Sciences and Nanosystems Ca’ Foscari University of Venice Venezia Italy; ^3^ Department of Chemical Pharmaceutical and Agricultural Sciences University of Ferrara Ferrara Italy; ^4^ Institute of Nanostructured Materials (ISMN) – National Research Council of Italy (CNR) Bologna Italy; ^5^ Department of Pharmacy and Biotechnology (FaBit) University of Bologna Bologna Italy; ^6^ IRCCS Istituto delle Scienze Neurologiche di Bologna Bologna Italy; ^7^ Department of Mechanical Engineering Chalmers University of Technology Gothenburg Sweden

**Keywords:** astrocyte calcium signaling, biodegradable electrodes, electrochemical neurotransmitter sensing, graphene‐based neural interfaces, laser‐scribed bioelectronics, PLA/graphene oxide composites, sustainable neurotechnology

## Abstract

Biocompatible and biodegradable electrode platforms were developed using laser‐scribed poly(lactic acid)/graphene oxide (PLA/GO) composites, prepared via an innovative waterborne dispersion method. Laser treatment enabled the formation of graphene/graphite‐like structures. By varying the fluence of the laser used, it was possible to modulate the physico–chemical properties of the conductive traces obtained, finally leading to materials characterized by tunable sheet resistance, chemical structure, and morphology. Thanks to the low charge transfer resistance and the peculiar electrocatalytic and antifouling properties observed, the platforms were applied as electrochemical sensors for the detection of various biomarkers in biological fluids, namely ascorbic and uric acid, nicotinamide adenine dinucleotide (NADH) and catecholamine‐based neurotransmitters, outperforming commercial carbon screen‐printed electrodes. Contextually, laser‐scribed electrodes were used to demonstrate the effectiveness of a novel approach for selectively stimulating Ca^2^^+^ signaling in astrocytes. The results here reported open the possibility to apply laser‐scribed PLA/GO as innovative eco‐sustainable solutions for simultaneous treatment of pathological conditions and monitoring of the resulting neurotransmitter expression in in vitro and in vivo models.

## Graphical abstract

This work introduces sustainable PLA/graphene oxide bioelectronic interfaces featuring electrodes with tunable conductivity and strong electrocatalytic performance. These platforms were used to implement a new method for tuning astrocyte Ca^2^^+^ signaling and for the efficient detection of key biomarkers. By combining green laser‐based fabrication, effective electrochemical sensing, and controllable neural stimulation, this study opens pathways toward next‐generation green neurotechnologies.

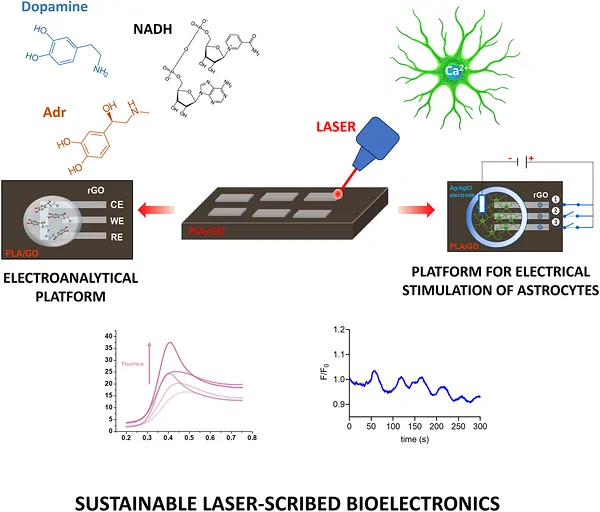

## 1 Introduction

Thanks to its lightness, strength, low cost, and ease of processing, plastic has gradually become part of human history, increasing the accessibility of goods and services and enabling significant advances in diverse fields such as transport, construction, medicine, and electronics. Evidence of plastic use can be traced back to the creation of rubber balls by the Mesoamericans around 1600 BC, while modern plastics emerged in the mid‐1800s with the production of semi‐synthetic plastics. Subsequent innovations such as Bakelite, PVC, cellophane, and nylon led to the mass production of plastic objects. Since the 1950s, global plastic output has grown exponentially, reaching 400 million metric tons in 2022. Despite their contribution to technological and societal progress, plastics generate escalating waste, projected to surpass 12 billion tons by 2050, posing severe environmental and public health risks [1, 2, 3]. Furthermore, technological advances have increasingly led to the integration of electrical circuits and devices into plastic products, improving their connectivity and functionality. These developments have exacerbated the problem of electronic waste and the associated issues of difficult recycling and potential toxicity [4].

The enhancement of the recyclability of e‐waste or the adoption of biodegradable systems, in conjunction with the implementation of more sustainable and environmentally friendly production methods, are effective solutions to contribute to a more sustainable future, in accordance with the European Green Deal and the United Nations Sustainable Development Goals. These approaches are also consistent with the One Health perspective, which recognizes that human well‐being is intrinsically linked to the health of the environment and ecosystem.

Polylactic acid (PLA) is a nontoxic, biodegradable, and recyclable polymer that can be derived from renewable sources. It is considered a promising, environmentally friendly, and biocompatible alternative to petroleum‐derived plastics. Currently, PLA is one of the most produced bioplastics in the world, widely used in packaging and biomedical applications. Eco‐friendly and multifunctional electronic platforms can be created by combining PLA with cutting‐edge manufacturing techniques, opening numerous potential applications in biomedicine, bioelectronics, intelligent food packaging, and sensors [5, 6, 7].

In recent years, the use of laser beams to induce localized pyrolysis of carbon‐based substrates has emerged as a new promising approach for creating graphene/graphite‐like structures starting from poorly conducting polymer films. This method has been extensively investigated with respect to different materials, laser sources, processing parameters, and experimental conditions [8, 9]. The laser functionalization process modifies the surface of composites, creating extended sp^2^ hybridized carbon structures. In contrast to the original polymer, these structures can have excellent electrical properties (sheet resistance of a few Ω sq^−1^), which allow this technique to be used to realize conductive tracks without the use of metal. This sustainable and scalable technology enables the design and fabrication of ad hoc devices, offering several advantages over other technologies such as the simplified production processes, fewer production stages and, with a proper selection of the plastic substrate, the possibility of improved recyclability and biodegradability, due to the reduction in the number of metal–plastic hybrid parts [10].

One of the approaches used for the treatment of polymers that alone cannot convert into conducting, graphene‐like structures by laser treatment is the inclusion of graphene oxide (GO) into the polymer matrix. The laser treatment induces the local conversion of sp^3^ carbon atoms into sp^2^ hybridization of GO and ablation of insulating materials, leading to a significant increase in electric conductivity. This approach was effectively used for the realization of conductive polyurethane platforms, applicable to produce electric circuits, electrochemical sensors and heaters [10]. Although polyurethane has considerable potential in the field of biomedicine due to its tunable properties, there are some aspects that need improvement, such as biodegradability, biocompatibility, safety risks, and sustainability [11]. The issue of sustainability assumes particular significance in the context of the production of disposable sensors and bioelectronic devices, due to the massive accumulation of waste.

Among advanced organic materials, poly(lactic acid)/graphene oxide (PLA/GO) composites have emerged as promising materials for interfacing with biological systems [12, 13]. This is due to the synergistic combination of biocompatibility and biodegradability of the polymers [14] with the good mechanical strength, flexibility, and ease of functionalization of graphene nanomaterials [13], offering enhanced stability and functional versatility for biomedical applications. This implies that the realization of conducting interfaces starting from PLA/GO can be applied to numerous biomedical applications, ranging from electric stimulation of neural cells to the definition of the chemical composition of biological fluids by electrochemical sensors.

In this work, we study the properties of novel electrodes fabricated by laser scribing PLA/GO films obtained starting from PLA particles synthesized by an innovative eco‐friendly procedure [15, 16] which does not require the use of toxic organic solvents. The devices developed were then applied to two potential cutting‐edge biomedical applications in neurotechnology: the electrochemical detection of biomarkers in biological fluids and the selective stimulation of astrocytes' Ca^2^^+^ signaling (Figure 1).

**FIGURE 1:**
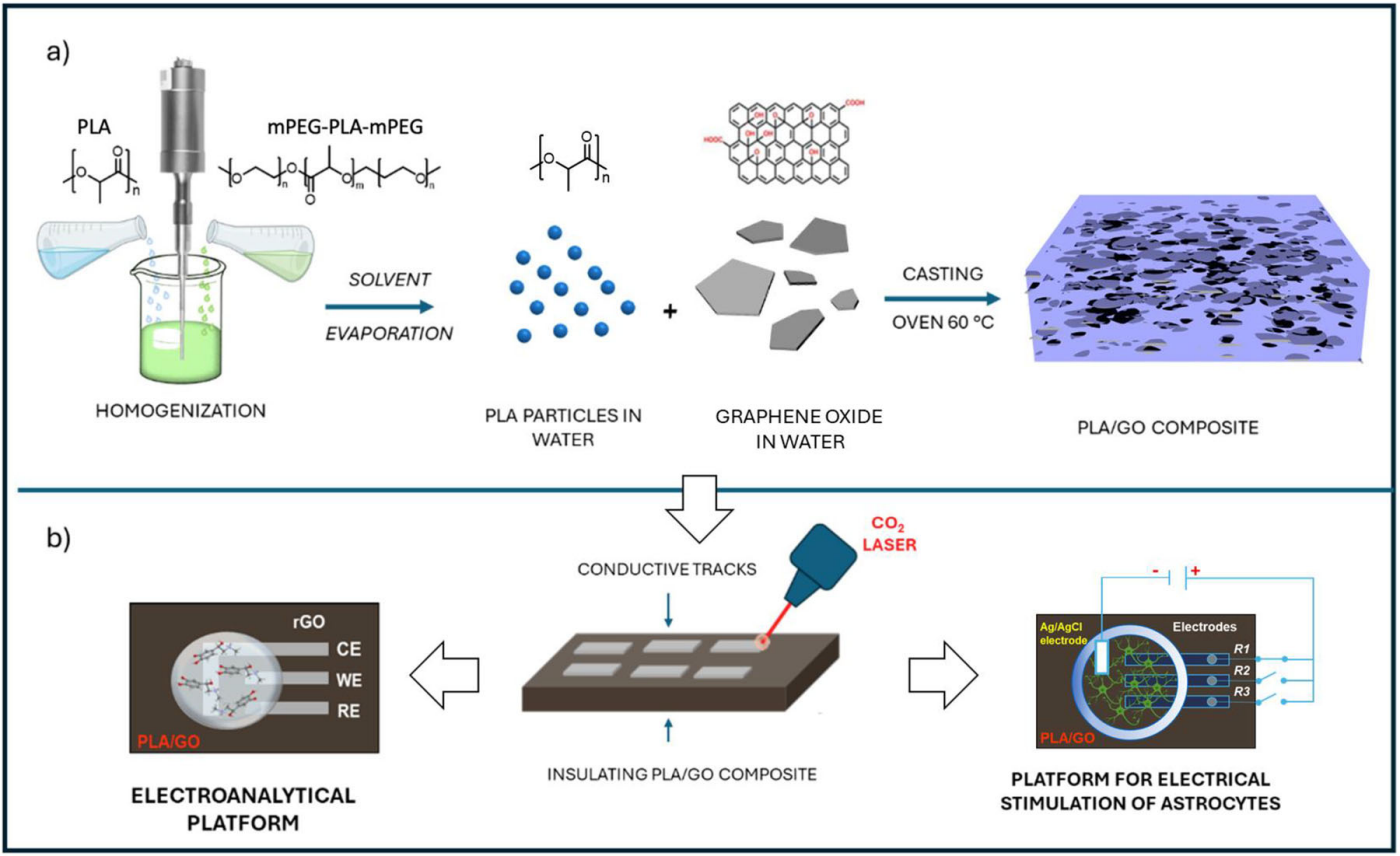
Schematic flow of the composite preparation (a) and devices fabrication (b).

In the first application, we exploit the electrocatalytic properties of the laser‐treated PLA/GO platforms for the detection of several molecules important for human health. The research was particularly focused on the detection of catecholamine neurotransmitters, which are important biomarkers of stress and neurological disorders that play essential roles in numerous biological functions. These tests allowed us to demonstrate that the proposed laser‐scribed PLA electrodes provide outstanding performance with respect to commercial carbon screen printed electrodes (CSPE), used as a benchmark. The correlation between the intensity of the laser fluence adopted for the realization of conductive traces and the physico–chemical properties of the resulting material allowed us to better understand the performance finally observed, which is essential for the detection of these species in biological fluids.

Contextually, in the second application, we explore the possibility of implementing the laser‐scribed PLA/GO platform as an electrode interface with the central nervous system.

Despite advances in neuroscience and bioelectronics, most current technologies remain focused on neurons, neglecting astrocytes [17, 18]. Astrocytes can sense and respond to various extracellular chemophysical changes (neurotransmitters, such as noradrenaline, adrenaline, dopamine, adenosine, glutamate etc., thermal, osmotic, mechanical, and electrical stimuli) through intracellular calcium ([Ca^2+^]_i_) dynamics [19, 20] and gliotransmitter release (such as glutamate, d‐serine, and ATP) [21, 22], playing a key role in brain health and disease. This evidence drives the development of advanced graphene‐based bioelectronic interfaces enabling dialogue with astrocytes [17, 23, 24].

In this work we verified that laser‐scribed PLA/GO electrodes support astrocyte adhesion and growth, confirming their suitability for application at the electrode‐brain tissue interface. We also demonstrated the efficacy of a novel method for the selective modulation of astrocyte Ca^2^^+^ signaling using laser‐scribed electrodes with different electrical resistances. This innovative signal modulation capability could provide the basis for advancing our understanding of astrocyte function and intercellular communication within the neural networks.

Overall, the results of this work suggest that PLA/GO platforms can be employed for future innovative solutions in laser‑scribed bioelectronics for the simultaneous treatment of pathological conditions and monitoring of the resulting neurotransmitter expression in in vitro and in vivo models.

## 2 Results and Discussion

### 2.1 Preparation and Characterization of PLA/GO Composite

A common method for producing PLA/GO composites is solution blending, a process that typically requires the use of toxic and environmentally harmful solvents [25]. The development of alternative methods in line with the Safe and Sustainable by Design principle, ensuring safer and more environmentally friendly processing by reducing hazardous solvents and minimizing the environmental impact of fabrication, is highly desirable. In accordance with this purpose, in this work, we exploit the use of an innovative waterborne dispersion of PLA recently developed for the preparation of the PLA/GO composite. This new eco‐friendly synthetic approach exploits the use of block copolymers of polyethylene glycol (PEG) and PLA (mPEG–PLA–mPEG) as surfactants, enabling the stabilization of polymer particles in an aqueous medium [15, 16].

Self‐standing PLA/GO films were prepared by solvent casting from a water dispersion of PLA particles and GO nanosheets at different concentrations (0.5%, 1%, and 2%, respectively), as schematized in Figure 1a and detailed in the Experimental Section. The films realized are hereafter referred as PLA/GO‐0.5%, PLA/GO‐1%, and PLA/GO‐2%, respectively. As a comparison, an analogous film not containing GO was also obtained in similar conditions named PLA/GO‐0%. The morphological, thermal, and biodegradation properties of PLA and PLA/GO composites were investigated using different techniques, including scanning electron microscopy (SEM), thermogravimetric analysis (TGA), differential scanning calorimetry (DSC), and biodegradation tests.

SEM images of the PLA/GO‐2% (Figure 2a) and PLA/GO‐0% (Figure 2b) film cross‐section reveal a compact structure, indicating good coalescence of PLA particles and the absence of macroscopic GO aggregates. This suggests an effective dispersion of GO within the PLA matrix at the micrometric scale. These observations are consistent with XRD analysis (vide infra), which shows no evidence of the characteristic GO diffraction peak associated with layered aggregates.

**FIGURE 2:**
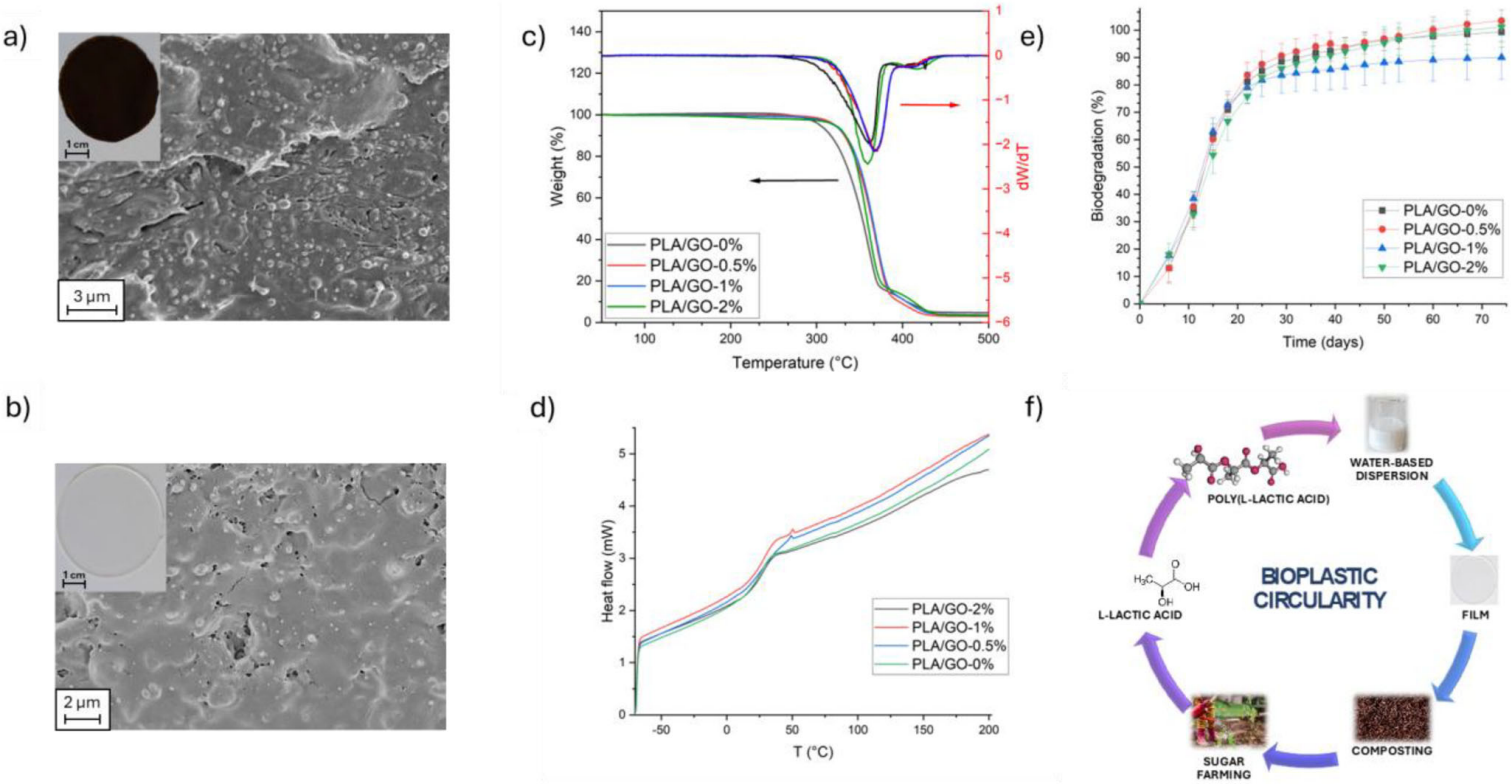
SEM images of the cross section of (a) PLA/GO‐2% and (b) PLA/GO‐0%; in the insets the optical images of the corresponding self‐standing films. (c) TGA curves of PLA/GO samples (below) and their first‐order derivatives (above). (d) Second heating DSC scans (endo up) of PLA/GO samples and (e) biodegradation curves in composting conditions of PLA/GO samples, error bars represent standard error (3 samples). (f) Life cycle of PLA.

The use of mPEG‐PLA‐mPEG block copolymers to stabilize PLA particles in water has the potential to enhance the interaction between PLA and GO, facilitating the dispersion of GO in the composite. This hypothesis is supported by previous studies, which demonstrated that the interaction between GO and PLA was enhanced by surface grafting GO with PEG [26].

Film formation from waterborne dispersions of preformed polymers is generally described as a three‐stage process consisting of drying, deformation, and coalescence. During drying, water evaporation increases the polymer volume fraction, leading to particle deformation and contact, followed by interdiffusion of polymer chains and formation of a continuous film [27].

Based on this mechanism, it can be hypothesized that the mPEG–PLA–mPEG surfactant, primarily located at the particle/water interface in the waterborne dispersion, may initially be positioned at the interface between PLA particles and graphene oxide, as well as between adjacent PLA particles during film drying. Particle packing during drying could promote the confinement of fillers within the interstitial regions between polymer particles, especially considering the much larger lateral size of GO flakes (up to several micrometers) compared to the PLA particles (∼400 nm).

Subsequently, particle coalescence and polymer chain interdiffusion during film formation may lead to a partial redistribution of the surfactant within the bulk polymer matrix. This is supported by the significant decrease in the *T*_g_ of the composites compared to the virgin polymer as observed in the DSC measurements (see discussion below in Section 2.1), indicating a plasticizing effect of mPEG–PLA–mPEG. Moreover, owing to its amphiphilic nature, the mPEG–PLA–mPEG copolymer is expected to interact with both PLA and GO, enhancing their interfacial compatibility and improving the dispersion of GO in the composite.

TGA (Figure 2c) evidenced a two‐step degradation behavior. The first, most pronounced step (derivative maximum at 360°C–370°C) is related to the PLA matrix. The second one (derivative maximum at 400°C–420°C), which is smaller and partially overlaps with the first, is related to the PEG segments, added as surfactant, which degrade at a higher temperature, as already observed in literature [28]. The addition of GO to the films caused three effects. First, a small weight loss is noticeable at temperatures < 300°C, that is, before the main degradation step begins. This is particularly evident in the samples with higher GO content and can be likely ascribable to the thermal removal of oxygen functional groups massively present in GO [29]. Second, the main degradation onset is shifted to higher temperatures by ∼10°C when GO is added. This indicates a slight thermal stabilization of PLA by GO that is already maximized at 0.5% GO concentration, as it does not increase at higher GO contents. As observed by several authors [30, 31, 32], this is likely due to the large surface area of the GO nanosheets creating barriers against the escape of volatile gases. On the other hand, the point of maximum degradation rate was shifted slightly upward (370°C vs. 362°C) when GO content was 0.5% or 1% but shifted again to lower temperatures (360°C) when the GO content reached 2%, meaning that with a further increase in GO the degradation reaction is actually accelerated despite its onset being delayed. A third effect is that the final residue is reduced from 4% to below 2% when GO is added, indicating a more efficient pyrolysis of the carbon residues when GO is present.

DSC analysis (Figure 2d) evidenced a single glass transition and no melting peaks due to the amorphous nature of the PLA used. The *T*_g_ is substantially lower than that of the virgin polymer (25°C–29°C vs. ∼60°C) due to the surfactant dissolved within the polymer matrix, which has a soft PEG block that acts as a plasticizer. The surfactant was completely miscible in PLA, as no separate glass transition related to the PEG component was observed. Increasing the quantity of GO added to the PLA films had minor effect on the glass transition behavior.

As already stated in the Introduction, PLA is a bioplastic that offers different ecological end‐of‐life options, from recycling, using mechanical or chemical processes, to industrial composting. Composting methods are an important opportunity to reduce the amount of waste sent to landfill as well as the methane emission and the overall global warming potential [33]. To verify this point, biodegradation tests on PLA/GO composites are reported in (Figure 2e). All PLA samples demonstrated rapid biodegradation, resulting in over 82% mineralization in industrial composting conditions after 32 days of observation. After this, the measured biodegradation of all samples continued to slowly increase over time, reaching values of over 90% for all samples after 74 days. No remains of the test samples were found in the containers after the test was completed; all of these materials are thus fully disintegrated and biodegraded in composting conditions.

Overall, no significant difference in compostability caused by GO addition or the surfactant presence into the films was observed: the sample with 1% GO had somewhat lower ultimate biodegradation values than the other samples but with low statistical significance (*p* > 0.1 at 74 days). The high compostability of all samples also proves that the presence of GO does not interfere with microbial activity. GO composites with biodegradable polymers have been previously shown to be capable of accelerating their hydrolytic and enzymatic biodegradation [34] but in our case the overall shape of the various biodegradation curves was very similar, attesting to no significant effect of GO on biodegradation rates of the material. GO itself is biodegradable and can be metabolized by soil microorganisms to CO_2_ and biomass in aerobic conditions [35].

PLA is considered a key sustainable material for replacing petroleum‐derived plastics due to its environmental benefits and properties. It can be obtained from renewable sources, such as cornstarch or sugarcane, and it is completely biodegradable. The products of its degradation, such as CO_2_ and water, could be absorbed and utilized by plants, creating a closed‐loop system (Figure 2f). The efficient biodegradation obtained for PLA/GO suggests that also this composite could be a promising material in bioplastic circularity.

### 2.2 Laser Patterning of PLA/GO Composites

Conductive pathways were formed by exposing PLA/GO composites containing different concentrations of GO (0.5%, 1%, and 2%) to a pulsed CO_2_ laser (Figure S1b,c). The increase in temperature caused by the laser treatment leads to the reduction of the GO and the degradation of the polymer, forming a network of conductive materials as detailed in the following sections. Figure S1d reports the electrical properties of the PLA/GO‐0.5%, PLA/GO‐1%, and PLA/GO‐2% composites as a function of the laser fluence tested (the effects of other laser parameters on the sheet resistance can be also found in the Supporting Information). After laser scribing, the sheet resistance of the three materials decreases by several orders of magnitude compared to the insulating starting composites. Conversely, samples without GO (PLA/GO‐0%), did not show any electrical conductivity even after laser treatment. The sheet resistance decreases as the laser fluence increases due to longer exposure times, which generally is correlated to an increase of the temperature of the laser‐radiated region [36]. The composite with a higher GO concentration (PLA/GO‐2%) exhibits appreciable sheet resistance (93 ± 16 Ω sq^−1^) even when treated at low laser fluence (0.59 J cm^−2^), a feature that is not found in other PLA/GO composites containing a lower amount of GO. The lowest achieved sheet resistance is 12 Ω sq^−1^ for PLA/GO‐2% (Fluence = 0.81 J cm^−2^), which corresponds to a conductivity of ∼21 S cm^−1^ assuming a thickness of sample modified by the laser of ∼40 µm (see later in the text). This value is comparable to that of laser‐induced graphene obtained from polyimide reported by Lin et al. [37].

The investigation of the surface of PLA/GO‐2% after laser treatment at different fluences (see SEM images in Figure 3 and Figure S3) reveals significant morphological changes once the material becomes conductive, that is, at a laser fluence of 0.59 J cm^−2^. The surface of this sample is characterized by the presence of numerous holes measuring up to tens of microns in size accompanied by raised flaps of material. Several droplet‐like structures are also visible, which are probably the result of polymer residue softened or decomposed during laser treatment. With an increase in laser fluence, the structures that appear as drops disappear, while the fractures increase along the laser scan direction, revealing a layered organization of the material (Figure S5a, SEM images in Figures 3 and S3). The layered structure is most evident in the cross‐section of laser‐treated materials (Figure S5b,c,d), which shows a morphology analogous to that of a GO film or GO polymer composite after laser treatment shown in different works [10, 38]. Similarly to what happens with these materials, the presence of space between layers may be due to the release of gaseous products during GO reduction or polymer degradation, which is caused by an increase in temperature resulting from laser irradiation. In particular, several studies have reported the release of gaseous products such as CO_2_ and CO during the decomposition of oxygen‐containing functional groups present in GO due to laser reduction or by thermal degradation of PLA [10, 39]. The hypothesis of degradation of PLA and the elimination of its oxygenated functional groups is supported by the EDX maps of carbon and oxygen of the cross‐section of the laser‐treated PLA/GO‐2% (see Figure S5e–h). The oxygen content of the PLA/GO composite (see the bottom part of Figure S5g) varies in the composite from ∼27% to ∼5% after laser treatment (see the upper part of Figure S5g).

**FIGURE 3:**
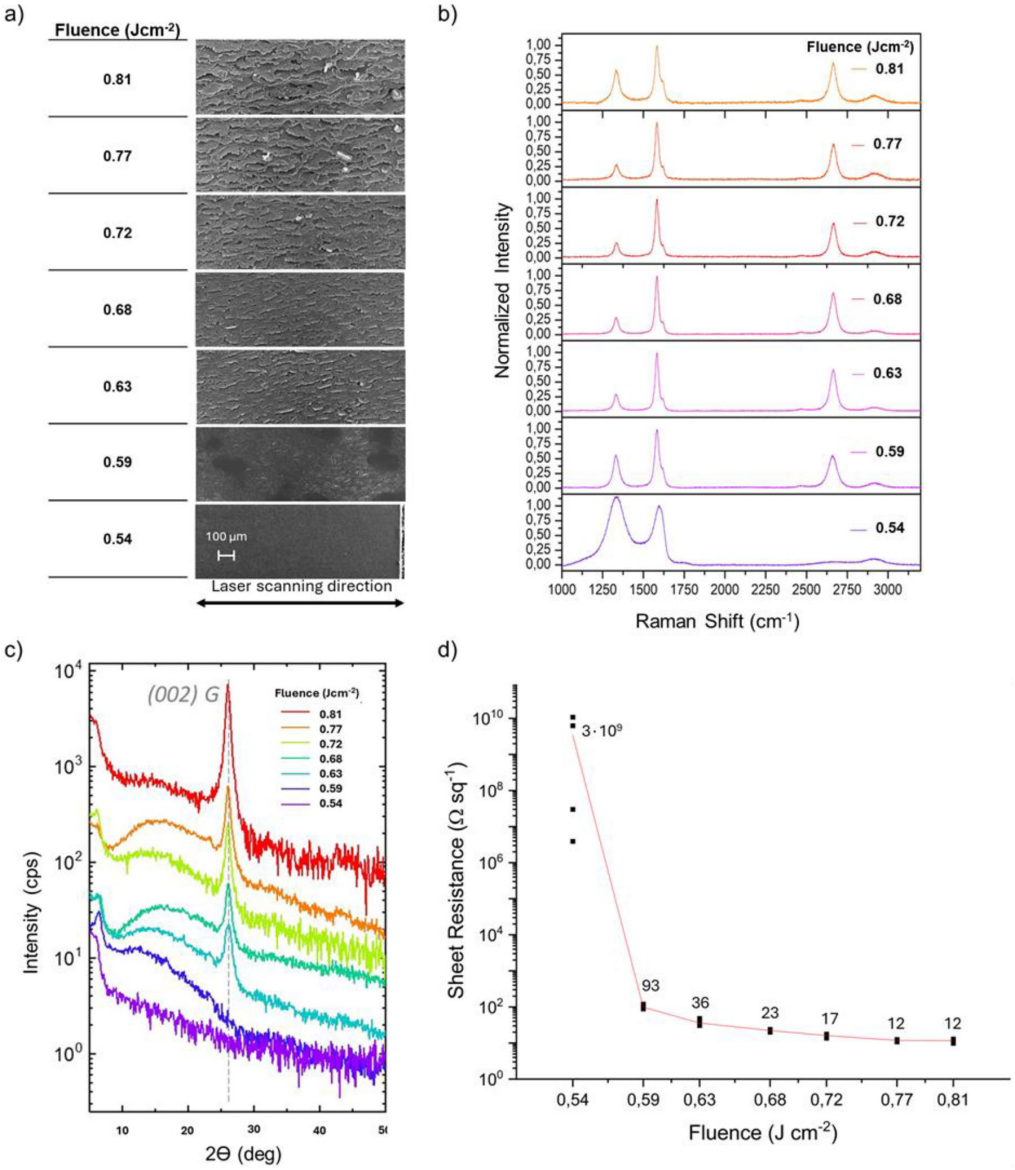
Characterization of PLA/GO‐2% after laser treatment at different fluences: (a) SEM, (b) Raman spectroscopy, (c) grazing incidence x‐ray diffraction and (d) sheet resistance. In (d), points represent individual measurements on 4–5 tracks from 2 samples for each fluence, while line and labels indicate the average values.

Optical images of the cross section (Figure S4) of PLA/GO‐2% treated with different laser fluences show that the thickness of the laser‐modified area increases with increasing laser fluence tested, reaching a maximum of ∼40 µm for a fluence of 0.81 J cm^−2^.

Raman spectroscopy indicates that laser treatment converts the PLA/GO‐2% composite into graphene/graphite‐like structures (Figure 3). The Raman spectrum of the sample treated with 0.54 J cm^−2^ laser fluence shows two intense peaks at 1332 and 1585 cm^−1^, which are attributable to the D and G peaks of GO. Increasing laser fluence on the sample causes a significant change in the spectrum with the decrease of *I_D_/ I_G_* ratio from 1.16 at 0.54 J cm^−2^ to 0.29 at 0.77 J cm^−2^ and the growth of an intense 2D peak at 2665 cm^−1^ characteristic of graphene/graphite‐like structures. The *I_D_/ I_G_* ratio decrease testifies to the effective reduction of GO and the decrease in the number of defects. The position of the 2D peak below 2700 cm^−1^ confirms the formation of few‐layers graphene in agreement with the literature [40]. The additional increase in laser fluence at 0.81 J cm^−2^ causes an increase in the *I_D_/ I_G_* ratio at 0.58. This indicates that laser treatment can create defects in material structures when the laser fluence is too high. Similar effects have been observed in literature [10, 41].

In line with SEM and Raman analysis, the XRD patterns evolve with laser fluence (Figure 3). In the PLA/GO‐2% film, no GO‐related reflections are detected (Figure S6a), consistent with a good dispersion of GO within PLA (absence of layered aggregates). The low‐angle feature at ∼6° originates from the pristine PLA matrix, as confirmed by specular *θ*–2*θ* scans of GO‐free PLA/GO‐0% film (Figure S6). Upon increasing the laser fluence, a diffraction peak appears at 2*θ* ≈ 26° (*d* ≈ 3.43 Å), which we assign to graphitic stacking (002) produced by laser writing [37]. The (002) signal becomes clearly resolved from 0.63 J cm^−^^2^ upward and increases monotonically with fluence, as captured by the (002) integrated area trend (Figure S6b). In parallel, the out‐of‐plane coherence length L_002_, estimated from the (002) line broadening using the Scherrer equation (Figure S6c), also increases with fluence, indicating that higher fluence not only increases the amount of graphitized material (area) but also improves the stacking order (larger L_002_). This provides a natural explanation for the apparent decoupling between sheet resistance and XRD at the lowest fluences: electrical percolation can occur once a connected network of conductive sheets forms even when stacked domains remain too short/disordered (small L_002_) to yield a detectable (002) reflection, whereas XRD requires sufficient out‐of‐plane periodicity. Consistently, the relative prominence of the (002)G peak increases with fluence; while only qualitative, this trend is compatible with progressive reduction/structuring of the GO component.

We propose an explanation of the laser transformation process in PLA/GO composites to better understand the mechanism behind these observations. When exposed to CO_2_ laser, PLA/GO converts light energy into thermal energy, promoting thermal degradation and graphitization reactions. CO_2_ laser irradiation is expected to generate instantaneous heating of GO [42] due to its high absorptivity in the infrared regions and transfer it to the surrounding PLA matrix. It is evident that the effects of laser treatment on the subject material had a significant impact on both surface morphology, structure and chemical composition. The heating process is expected to convert insulating GO into electrically conductive reduced graphene oxide (rGO) by removing its oxygen functional groups and partially restore the percolation pathways of conductive aromatic carbon in the basal plane of GO [10, 42]. Simultaneously several processes could occur for PLA, including softening, thermal degradation and ablation of polymer with the formation of gaseous products that leave the matrix [39, 43, 44].

Taken together, the experimental results obtained from electrical measurements, SEM/EDX, Raman, and XRD analyses support this interpretation. Briefly, as reported above, SEM/EDX reveals a layered morphology of the laser‐treated material analogous to that of laser‐reduced GO described in literature, accompanied by a significant decrease in oxygen content and the presence of void regions consistent with polymer ablation, release of gaseous products, and concurrent GO reduction. Electrical measurements demonstrate high conductivity, while Raman and XRD confirm the formation of low‐defect graphene‐like structures and graphitic stacking consistent with reduced graphene oxide.

To further clarify the role of GO in laser‐treated PLA/GO composites, additional control experiments were performed under identical laser‐processing conditions using PLA/GO‐0%, films with only GO (GO‐100%), and PLA composites containing alternative fillers. PLA/GO‐0% was used to evaluate the intrinsic response of the polymer matrix to laser irradiation, while GO‐100% films allowed assessment of the direct response of GO in the absence of the polymer matrix. PLA composites containing BN or SiO_2_ fillers were investigated as additional reference systems to assess whether laser treatment could induce the formation of conductive structures through PLA carbonization in the presence of fillers other than GO (see Supporting Information for more information).

Laser treatment of PLA/GO‐0% did not result in the formation of conductive tracks. At higher laser powers, the irradiated regions were almost completely depleted of material, leading to complete removal of the material and formation of through‐holes having the shape of the entire laser‐written path (Figure S2a). Similar to PLA/GO‐0%, PLA composites containing BN or SiO_2_ fillers did not develop conductive structures after laser treatment. In contrast, GO‐100% films consistently yielded conductive tracks with low sheet resistance (∼20 Ω sq^−^^1^), comparable to those obtained for PLA/GO composites processed under identical laser conditions (∼17 Ω sq^−^^1^) (Figures S2b and 3d).

While these control experiments do not allow an unambiguous chemical identification of the carbon source (partial graphitization of PLA during the laser treatment of PLA/GO membranes cannot be completely excluded), they clearly demonstrate that the formation of conductive graphitic structures is contingent on the presence of GO under the processing conditions investigated. In particular, the comparison between GO‐100% films and PLA/GO samples provides strong evidence for the central role of GO in the formation of the conductive phase, indicating that GO itself can be directly converted into a highly conductive structure under the applied laser‐processing conditions. The PLA/GO composite illustrates the synergistic roles of its two components. The polymer matrix provides mechanical support, preserving the integrity of the conductive tracks during laser processing, whereas pure GO films tend to crack or fragment (see Supporting Information). In turn, GO promotes the formation of conductive structures and helps prevent complete ablation of the composite within the range of laser irradiation conditions explored in this work (see Figure S2a).

Biodegradation testing of laser‐scribed PLA/GO‐2% was carried out to test the continued compostability of the film after the process, by comparison with the original PLA/GO‐2% film. The results are shown in Figure S7, and show no significant difference between the two samples, confirming that the treated films remain fully biodegradable.

### 2.3 Application of PLA/GO in Electrochemical Sensors

The capability of PLA/GO‐2% based platforms to be used as electrochemical sensors was first evaluated using redox active benchmark species, namely 1,1‐ferrocenedimethanol—Fc(OH)_2_—and the ferro‐/ferri‐cyanide redox couple (FeCN^3+/2+^). The electrochemical behavior was compared to that of a more conventional electrode system, namely a commercial CSPE.

Figure 4a shows representative cyclovoltammetric (CV) responses recorded in 0.1 M KCl solution in the absence and in the presence of 0.5 mM Fc(OH)_2_. Similar traces were collected for PLA/GO‐2% platforms obtained at different laser fluences and at different scan rates (see Figure S8a–c).

**FIGURE 4:**
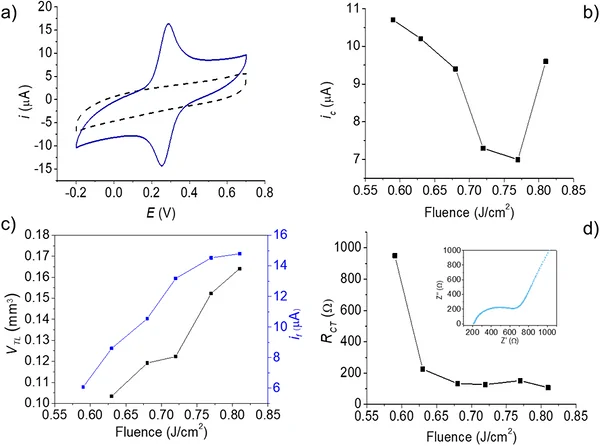
(a) CV responses recorded using an electrode made of PLA/GO‐2%, 0.72 J cm^−2^ laser fluence, in 0.1 M KCl in the absence (dotted line) and in the presence (solid line) of 0.5 mM Fc(OH)_2_; potential scan rate was 0.05 V s^−1^. Relevant trend of (b) capacitive current (*i*_c_) recorded in the absence of Fc(OH)_2_ at +0.27 V, (c) faradic current (*i*_f_) and volume of thin‐layer (*V*_TL_) recorded in the presence of Fc(OH)_2_. (d) Trend of (charge transfer resistance) *R_CT_* defined by EIS plots recorded in Fe(CN)_6_^2+/3+^ in 0.1 M KCl; an exemplificative response collected with PLA/GO‐2% at fluence of 0.72 J cm^−2^ is reported in the inset.

The capacitive current (*i*_c_), generated at the electrode‐solution interface by polarizing the electrode at a variable potential, was determined in the absence of faradic processes, sampling the current at 0.27 V. A progressive decrease of *i*_c_ was observed at increasing laser fluence (Figure 4b), reaching a minimum at 0.77 J cm^−2^. Interestingly, a further increase in fluence (0.81 J cm^−2^) led to a rise in *i*_c_. This trend aligns with Raman spectra, which suggests that higher laser fluences promote the formation of structural defects in the graphitic material. Such defects can enhance surface charge density, thereby increasing the capacitive contribution.

CV responses in the presence of Fc(OH)_2_ show the typical shape expected for a reversible redox species undergoing oxidation at a conductive electrode surface (Figures 5a and S8). The difference between the oxidation and the reduction peak potentials, in any case lower than 100 mV, indicates that PLA/GO‐2% platforms can be used as electrodes after the laser treatment regardless of the laser fluence applied. A more straightforward evaluation of the charge transfer resistance (*R_CT_*) was achieved by electrochemical impedance spectroscopy (EIS), performed using the FeCN^3+/2+^ redox couple (Figure 4d). The EIS plot indicates the presence of charge transfer and diffusive processes as expected for this redox couple on an electrochemically inert conductive surface. As observed, the *R_CT_* values decrease at increasing laser fluence, reaching minimum values at 0.72 J cm^−2^. Except for the platform obtained at the lowest laser fluence (0.59 J cm^−2^; *R_CT _*_=_ 950 Ω), the values obtained for all the electrodes tested are lower than that obtained with a commercial CSPE (*R_CT_* _=_ 470 Ω).

**FIGURE 5:**
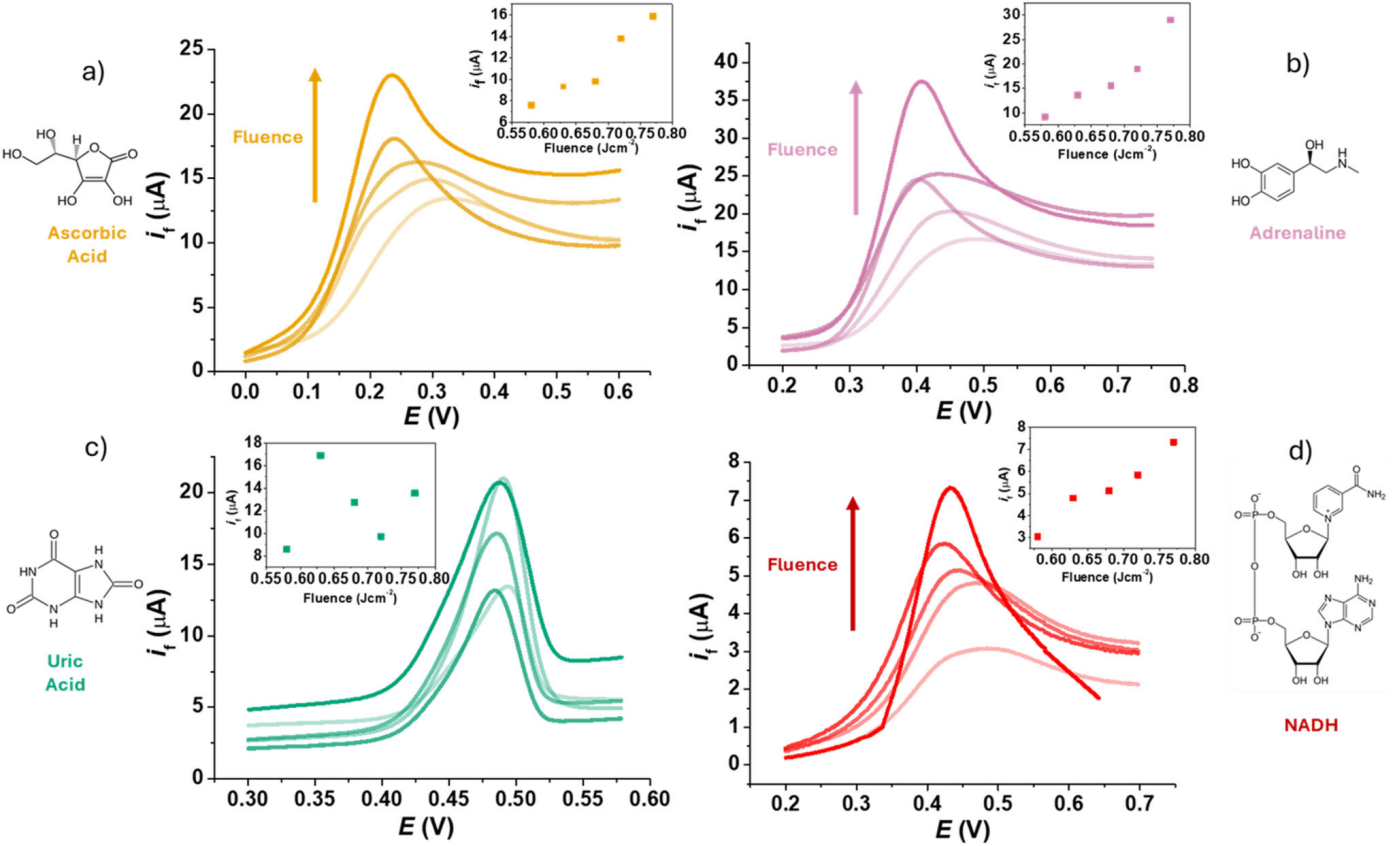
Linear sweep voltammetry oxidation responses recorded with PLA/GO‐2% platforms at various laser fluences in artificial sweat solutions also containing 1 mM (a) ascorbic acid, (b) adrenaline, (c) uric acid, and (d) NADH at 0.02 V s^−1^ potential scan rate. The curves are subtracted for the relevant blank signal.

The dimension of the electroactive surface in platforms obtained in various conditions was evaluated by considering the faradic contribution to the oxidation peak (*i*_f_) of Fc(OH)_2_ recorded in CV traces. As observed in Figure 4c, *i*_f_ increases with increasing laser fluence, reaching a plateau at approximately 0.77 J cm^−2^. This trend suggests a correlated increase of the electroactive surface until reaching a threshold. Quite interestingly, only voltammetric responses recorded with PLA/GO‐2% obtained at 0.59 J cm^−2^ (Figure S8a) exhibit the features of diffusion‐controlled processes, which are typical of flat conductive surfaces like CSPE (Figure S8d): a linear correlation between the peak current (*i*_f_) and the square root of the potential scan rate (*ν* ^1/2^) and the asymmetric peak shape characterized by a slow decay of the current after the maximum [45]. These conditions allowed us to calculate the electroactive area by the application of the Randles–Ševčík equation for the electrode laser‐treated at 0.59 J cm^−2^. Considering the slope obtained in this case (1.53 ± 0.03 mA s^1/2^ V^−1/2^), we could calculate an electroactive area of 10.21 ± 0.20 mm^2^, that is, a value larger than the nominal geometric one (9 mm^2^).

On the contrary, CV traces recorded with platforms obtained with higher laser fluences exhibit features typical of thin layers or of adsorption processes: a deep decrease of the current values after the oxidation peak and a linear dependence between i_f_ and the scan rate (Figure S8b,c). This peculiar behavior, rarely observed at bare electrode surfaces, can be explained by considering the morphological features of these surfaces defined by SEM images (see Figure 2), which suggest the porous nature of the electrode platform. Based on these results, the charge of the faradic process involving Fc(OH)_2_ oxidation was used to calculate the volume of thin‐layer (*V*_TL_) [46] for platforms obtained with fluence from 0.63 J cm^−2^ onward, that is the volume effective for charge transfer processes. As shown in Figure 4c, *V*_TL_ increases with increasing laser fluence, indicating an advantage in increasing the laser fluence to obtain electrode platforms characterized by higher sensitivity. From the value of the volume obtained at the various laser fluences (ranging from 0.103 to 0.167 mm^3^) and considering, for simplicity, that the electroactive area is not significantly affected by the laser fluence, a total thickness ranging from 10.0 to 16.3 µm was calculated as the space in which electroactive species can diffuse to undergo an effective charge transfer process with the conductive electrode surface. This is also in agreement with the optical images collected from the cross sections (Figure S4), showing that the laser fluence affects the PLA/GO‐2% at different depths.

The electrocatalytic properties of the laser‐treated PLA/GO‐2% platforms, which are at the basis of the use of these devices as electrochemical sensors, were investigated using electroactive biomolecules relevant to health monitoring. In particular, we tested adrenaline (Adr) and nicotinamide adenine dinucleotide (NADH), which are important biomarkers of neuronal stimuli, as well as ascorbic and uric acids, normally present in biological fluids (Figure 5). The increase of the laser fluence results in electrode platforms characterized by effective electrocatalytic properties, as evidenced by the shift of the current peak to less positive potential observed for ascorbic acid, Adr and NADH for laser fluences increasing from 0.59 to 0.77 J cm^−2^. The arising of electrocatalytic processes, normally due to oxidized moieties present on carbon‐based surfaces [47], allows the detection of these analytes at less positive potentials and with sharper oxidation peaks, thus improving the sensor selectivity and sensitivity, respectively. Indeed, a correlation between the intensity of the anodic peak and the laser fluence was observed for these molecules (Figure 5a,b,d), resulting from a combinatorial effect of the enhancement in electron transfer kinetics due to the electrocatalytic performance and the increase in the *V*_TL_ previously described. Vice versa, no correlation was observed for uric acid (Figure 5c), except for the platform obtained at the lowest laser fluence, that is, 0.59 J cm^−2^, which led to the oxidation of this species at higher potentials and with a broader peak, probably due to the higher *R_CT_* characterizing this platform. The poor correlation obtained in this case between the laser fluence and the electrocatalytic properties can be ascribed to the fouling of the electrode surface due to massive adsorption of this species. This is also confirmed by the asymmetric bell‐shaped oxidation peak [48, 49].

Based on the promising results observed for Adr oxidation and due to the importance of the detection of this species for the analysis of neural stimuli, we decided to test the applicability of PLA/GO‐2% as a sensor for the catecholamine‐based neurotransmitters involved in Adr biosynthesis, namely Tyrosine (Tyr), L‐DOPA, Dopamine (Dop), Noradrenaline (Nor), and Adrenaline (Adr). The electrochemical sensors obtained with a fluence of 0.72 J cm^−2^ were selected for these tests. Preliminary CV responses (Figure S9) revealed that all the molecules involved in the synthetic process of Adr can undergo electrochemical oxidation at PLA/GO‐2%, indicating that they can be detected by this sensor system. Quite interestingly, if compared with analogous responses collected at CSPE, a higher reversibility degree can be observed by using these innovative platforms, as typically obtained thanks to the arising of effective electrocatalytic processes.

Further voltammetric tests were carried out in differential pulse voltammetry (DPV), which is the voltammetric technique most frequently used for electroanalytical purposes. In analogy with the results obtained in CV, the DPV responses are shifted at lower potential values and show a higher peak intensity compared to responses recorded at CSPE (Figure 6a–f). In all cases, these preliminary investigations indicated a good correlation between the current peak and the concentration of the analyte in solution. This is due to negligible fouling or memory effects characterizing PLA/GO‐2% platforms, especially if compared to a different carbon‐based platform, namely CSPE (Figure S11). This fact evidences the advantages implied in the application of PLA/GO‐based electrodes for the detection of all these species involved in the biosynthetic pathway of Adr. The oxidation peaks observed are 0.737 ± 0.002 V for Tyr, 0.361 ± 0.007 V for L‐DOPA, 0.315 ± 0.005 V for Dop, 0.351 ± 0.010 V for Nor and 0.378 ± 0.009 V for Adr (Figure 6f), indicating the possibility to unravel the response of Adr oxidation with respect to those of the relevant precursors [50] that may be present in the same sample.

**FIGURE 6:**
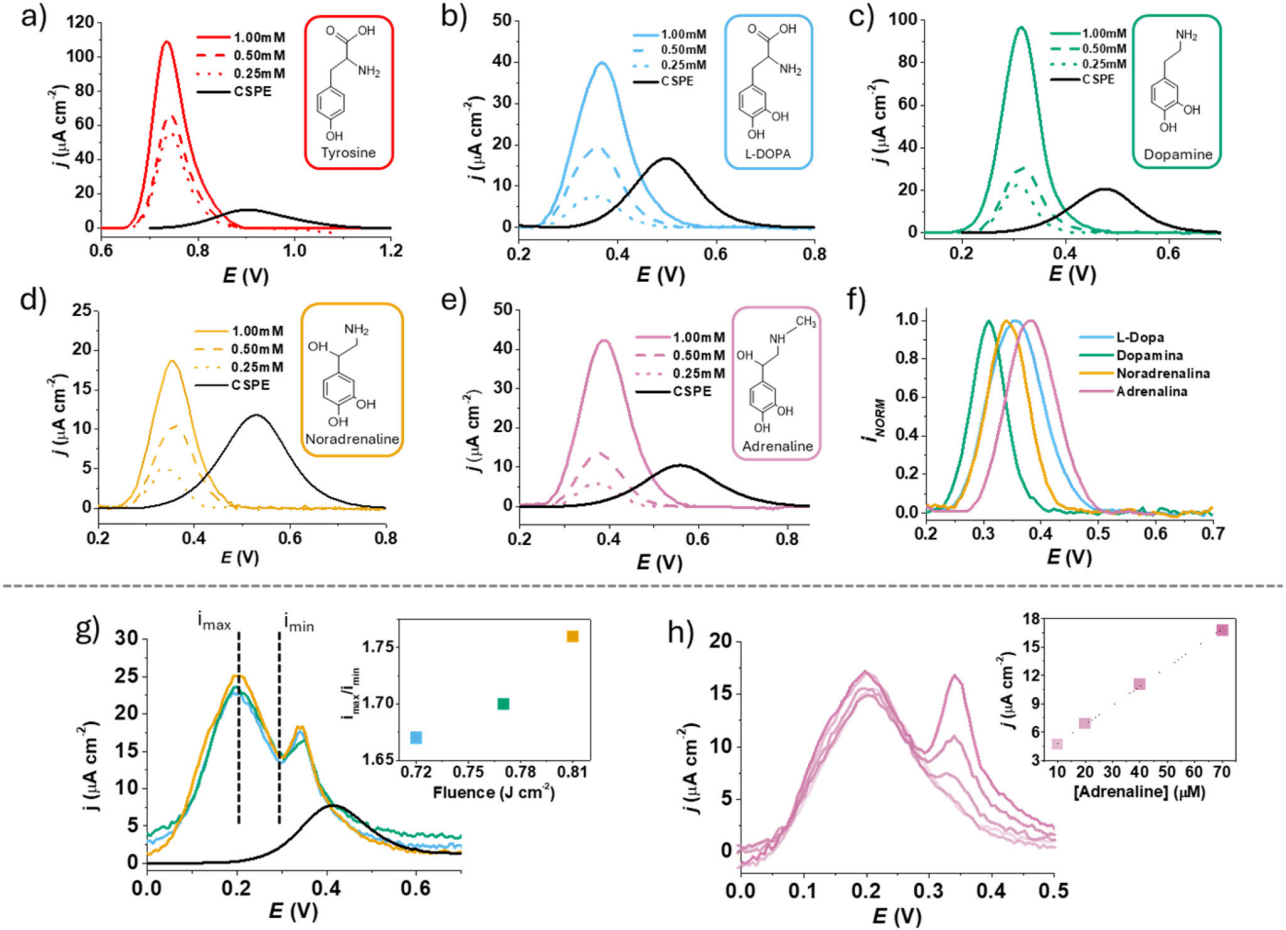
DPV responses recorded at PLA/GO‐2% in artificial sweat of three different amounts of the various molecules involved in the biosynthesis of adrenaline: Tyr (a), L‐DOPA (b), Dop (c), Nor (d), and Adr (e) compared to responses obtained in solutions containing the analyte at the highest concentration at a CSPE (black curve); (f) normalized DPV responses recorded for L‐DOPA, Dop, Nor, and Adr at PLA/GO‐2% to show the separation among the oxidation peaks. (g) DPV responses recorded with PLA/GO‐2% (0.72, 0.77, and 0.81 Jcm^−2^ fluence) in artificial sweat containing 50 µM Adr and 500 𝛍M ascorbic acid; the inset reports the *i*_max_/*i*_min_ vs. the fluence applied in the realization of the platforms; (h) DPV responses recorded in artificial sweat solution containing 0.5 mM ascorbic acid and different amounts of Adr (10, 20, 40, and 70 𝛍M); the inset reports the trend of anodic peak current recorded at 0.33 V vs. Adr concentration.

Subsequently, we focused the analysis on the detection of Adr. First, we evaluated the sensor's performance in terms of repeatability and reproducibility by performing three calibration plots from 0.1 to 1 mM with the same sensor platform and by repeating this analysis of three PLA/GO electrodes obtained with the same laser fluence, namely 0.72 J cm^−2^. The relative standard deviation of the sensitivity obtained in these conditions, 4.4% and 11.2%, respectively, indicates the very good repeatability and reproducibility of the sensor tested. These values are lower than those obtained in similar conditions with commercial CSPEs (5.8% and 17.7%, respectively), indicating enhanced measurement stability. Overall, the results demonstrate that the developed platform provides more reliable performance compared to standard commercial electrodes, particularly in terms of long‐term operational stability and batch‐to‐batch consistency.

Finally, the sensing performance for Adr detection was assessed in the presence of a common interfering species present in sweat, namely ascorbic acid, to verify the selectivity of the platform. A mixed solution containing 50 µM Adr and 500 µM ascorbic acid was tested with PLA/GO‐2% platforms fabricated at three different fluences, namely 0.72, 0.77, and 0.81 J cm^−2^. The results obtained were once more compared with analogous responses obtained at the CSPEs (Figure 7g). Besides the analysis of the peak intensity, the capability of the electrochemical sensors to achieve good resolution between the two oxidation processes was quantified by the ratio between the maximum current recorded for ascorbic acid oxidation (*i*_max_) and the current recorded in the minimum between the two oxidation processes (*i*_min_). As observed, although all PLA/GO platforms tested demonstrated good resolutions between the two responses, better performance was obtained from the platform obtained with the highest fluence. Conversely, CSPE shows no capacity to discriminate between Adr and ascorbic acid oxidation responses.

**FIGURE 7:**
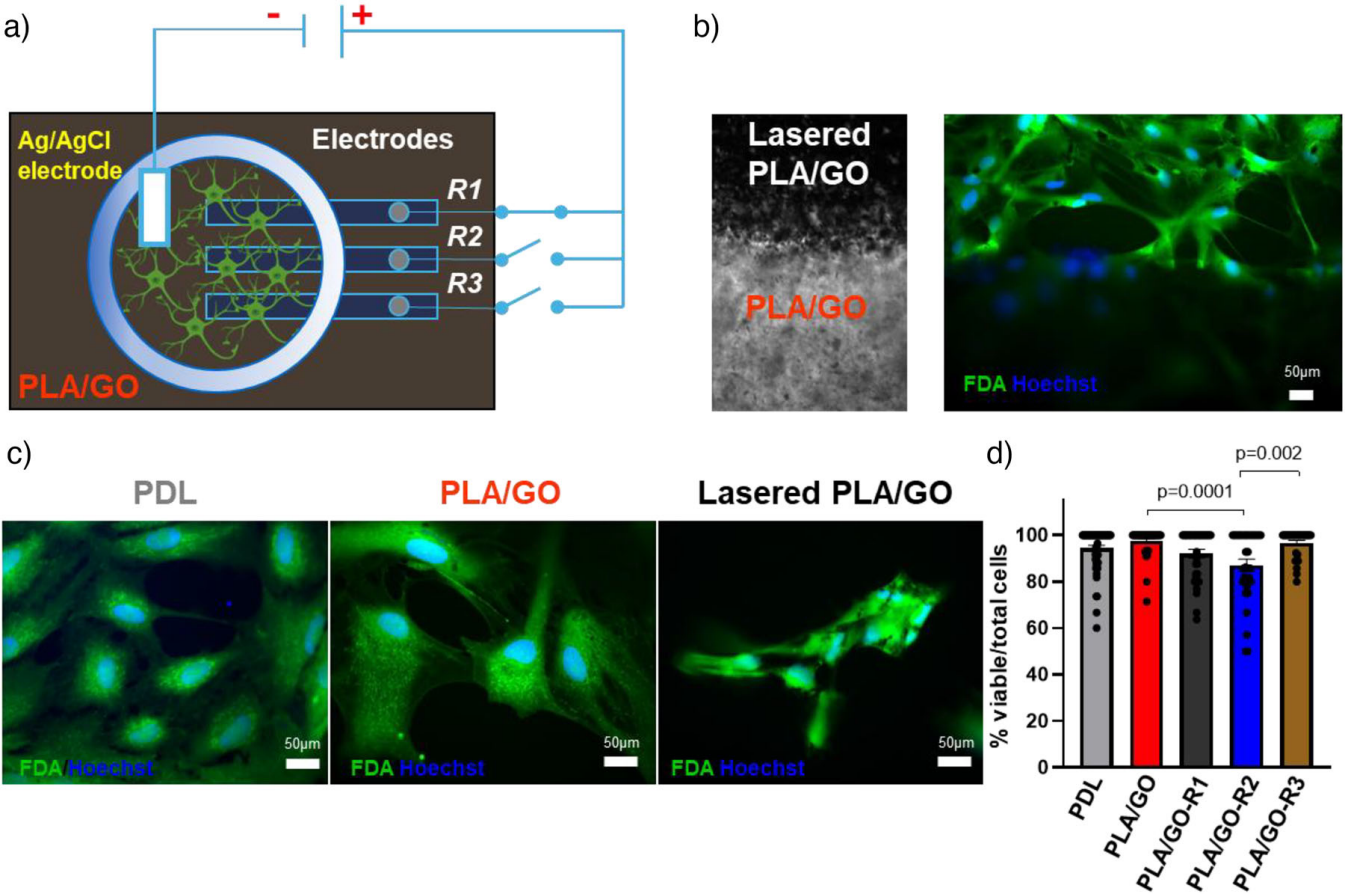
Laser‐scribed PLA/GO‐0.5% platform for electrical stimulation of astrocytes. (a) Scheme of the experimental set‐up for the electrical stimulation of astrocytes. (b and c) Fluorescence images of FDA/Hoechst‐stained astrocytes cultured on the laser‐scribed PLA/GO‐0.5% platform, shown at low (b) and high (c) magnification. Astrocytes cultured on poly‐D‐lysine (PDL)‐coated glass are shown as the standard culture control (c, left panel). (d) Statistical analysis of astrocyte viability on PLA/GO‐0.5% platform. For PDL, *n* = 56, *s* = 6, *N* = 3; for PLA/GO, *n* = 38, *s* = 6, *N* = 3; for PLA/GO‐R1, *n* = 34, *s* = 6, *N* = 3; for PLA/GO‐R2, *n* = 32, *s* = 6, *N* = 3; for PLA/GO‐R3, *n* = 29, *s* = 6, *N* = 3 (*n* = number of images analyzed, *s* = number of samples, *N* = number of experiments). Data are presented as mean ± s.e.m. Statistical significance was calculated via one‐way ANOVA with Bonferroni post‐test.

Given the promising results obtained for the detection of Adr even in the presence of ascorbic acid, we recorded the responses in solutions at different concentrations of analyte (0, 10, 20, 40, and 70 µM) and a fixed concentration of the interfering species (0.5 mM) to assess the lack of interference between the two species. As observed from DPV responses recorded in the various solutions (Figure 7h), the current associated with ascorbic acid oxidation remains constant regardless of the concentration of Adr and the current peak due to Adr oxidation still maintains a linear trend with the concentration. These results demonstrate that ascorbic acid does not interfere with the electrochemical detection of Adr.

Thanks to the biocompatible nature of the developed platforms (vide infra), combined with the excellent response repeatability and reproducibility, we verified the possible realization of an integrated sensor platform consisting of a 3‐electrode cell fully realized by laser scribing; in addition to the working electrode previously described, two further conductive traces were included, treating the PLA/GO surface with the laser (0.72 J cm^−2^) to obtain the counter and the reference electrode. The latter one was then coated with silver paste, to fix its potential to the Ag/AgCl redox couple. This prototype was used for Adr detection in artificial sweat solutions by recording DPV responses at different concentration (Figure S10a). The same analysis was repeated three times on three similar devices, collecting, at the end, nine calibration plots. A linear response was observed between 40 and 700 µM, yielding a sensitivity of 55.4(±2.0) µA cm^−2^ mM^−^^1^ and a limit of detection (LOD), calculated from the intercept of the calibration plots, of 4.5 µM. In these same experimental conditions, CSPEs displayed a sensitivity of 16.8(±1.1) µA cm^−2^ mM^−^^1^ and an LOD of 11.1 µM (Figure S10b). The PLA/GO platform thus exhibits a 3.3‐fold higher sensitivity together with an LOD 2.5‐fold lower with respect to commercial carbon‐based platforms, demonstrating better analytical performance for target detection.

Although higher sensitivities were reported for laser‐induced graphene‐based Adr sensors [51], the main advantage of the platform developed in this work is the intrinsic sustainability deriving from the use of a biodegradable material as an electrode support. In this respect, it is worth underlining that this first prototype of 3‐electrode cell represents a proof of concept of the possible realization of eco‐compatible sensor devices, offering clear advantages in terms of sustainability and operational cost that may make it preferable for disposable devices. The geometry and the overall setup of the device can be very simply adapted to the specific application sought and optimized to enhance the analytical performance of the device.

This notwithstanding, to further investigate the antifouling properties of the laser‐treated PLA/GO‐2% platforms also in this complete configuration, we quantified the variation of the faradaic oxidation peak for the 0.1 mM Adr solution during the three consecutive calibration plots (see Figure S11a,c). In addition, the variation in the *R_CT_* before and after three calibrations was assessed by EIS analysis in [Fe(CN)_6_]^3‐/4−^ solution. The results were still compared with the behavior of the commercial CSPE (Figure S11b,d). We found that CSPE highlights a pronounced decrease of the oxidation peak (‐42.0%), together with a positive shift of the process to more positive potentials (+0.095 V), while the laser‐treated PLA/GO exhibits a better current stability (−10.5%) and no significant variation of the oxidation potential (Figure S11a,c). This different behavior is consistent with the *R_CT_* increase observed for the commercial devices (+39.7%) compared to a nonsignificant difference observed for laser‐treated PLA/GO platforms, which indicates a better resistance to surface passivation. According to previous results [52] the massive fouling observed for CSPE can be associated with the formation of polyepinephrine, since the operating conditions are expected to promote the electropolymerization of epinephrine (i.e., Adr). Overall, the results demonstrate that the developed platform is less prone to electrode fouling than standard commercial electrodes.

These preliminary tests highlighted the potential of using laser‐treated PLA/GO for the development of electrochemical sensors that can be advantageous for on‐chip or in situ measurements. Overall, the devices obtained are well‐suited to monitor key neurotransmitters, such as Adr and other relevant catecholamines involved in its biosynthetic pathway, possibly involved during astrocyte stimulation experiments, providing a useful tool to investigate neuromodulatory effects and astrocytic metabolic responses, with significant implications for neuroscience research and bioelectronic medicine.

### 2.4 Platform for Electrical Stimulation of Astrocytes

In a previous work, we demonstrated that electrical stimulation using ITO electrodes coated with GO and rGO elicits distinct Ca^2+^ dynamics in astrocytes, in vitro and in brain slices. Specifically, ITO/GO stimulation evokes a slow, sustained calcium increase (“S‐type”), mediated by external calcium influx. In contrast, ITO/rGO stimulation induces rapid calcium oscillations (“P‐type”), exclusively driven by calcium release from intracellular stores. In our bioelectrical model, we hypothesized that the insulating/conductive properties of the substrate influence the electric field at the cell‐material or cell‐electrolyte interfaces, respectively, activating different intracellular signaling events [24]. However, there remains a critical need for advanced tunable platforms capable of selectively stimulating astrocyte calcium activity while simultaneously providing sensitive and precise monitoring of their signaling dynamics and chemical molecules in physiological and pathological environments.

In this study, we exploit the tunable sheet resistance of laser scribing of PLA/GO‐0.5% to selectively control astrocyte Ca^2+^ signaling in vitro through electrical stimulation (Figure 7a). The PLA/GO‐0.5% formulation was selected because it exhibits a broader range of sheet resistance achievable as a function of laser fluence than PLA/GO‐2%, enabling the fabrication of electrodes with more markedly different electrical properties. This wider modulation range allowed a more systematic investigation of the correlation between electrode properties and astrocytic Ca^2^^+^ responses. Based on these considerations, astrocyte stimulation experiments were performed on PLA/GO‐0.5% platforms containing 3 laser‐scribed electrodes (*R1, R2, R3)* with the same geometry but different mean electrical sheet resistances: 70 Ω sq^−1^ (*F *= 0.72 J cm^−2^), 150 Ω sq^−1^ (*F* = 0.68 J cm^−2^), and 600 Ω sq^−1^ (*F *= 0.63 J cm^−2^).

As a first step, to assess the biocompatibility of the laser‐scribed PLA/GO‐0.5% platform, primary cortical astrocytes were cultured on its surface [24, 53]. After 5 days in vitro, fluorescence imaging was performed using fluorescein diacetate (FDA) and Hoechst 33342 to visualize cytoplasmic and nuclear morphology, respectively. As shown in Figure 7b,c, viable astrocytes, identified by green FDA‐positive cytoplasmic fluorescence and blue Hoechst‐positive nuclear staining, adhered to both the laser‐scribed PLA/GO electrodes and the untreated PLA/GO substrate. Astrocytes grown on PLA/GO maintained a polygonal morphology comparable to that typically observed on the standard poly‐D‐lysine (PDL)‐coated glass. Notably, cells grown on the laser‐scribed electrodes appeared to establish a closer interaction with the material surface, tightly enwrapping the electrode microstructure.

Statistical analysis of cell viability, expressed as the percentage of FDA‐positive cells relative to the total Hoechst‐positive cell population (%viable/total cells, Figure 7d), revealed a high viability across all PLA/GO‐based substrates and laser‐scribed electrodes. Specifically, the % of viable cells on PLA/GO was comparable to that observed for astrocytes cultured on PDL‐coated glass. Compared with PLA/GO, a reduced cell viability was observed on the electrode with intermediate sheet resistance (*R*_2_), whereas cell viability significantly recovered on the electrode with higher sheet resistance (*R*_3_). This trend does not appear to correlate directly with variations in the electrical conductivity. Collectively, these results indicate that the material composition and surface laser patterning of the PLA/GO platform effectively support astrocyte adhesion and growth, confirming its suitability for application at the electrode‐tissue interface.

Next, the functionality of the laser‐scribed PLA/GO devices was evaluated by testing their ability to electrically stimulate astrocyte Ca^2+^ dynamics [24, 53, 54]. Electrical stimulus was applied by ramping up substrate voltage using an external Ag/AgCl reference electrode immersed in the same saline solution as the sample, in accordance with a previously established protocol [24, 53, 54]. Each laser‐scribed electrode, defined by a distinct electrical sheet resistance, was independently connected to the external voltage source to deliver a localized electrical stimulation (Figure 8a). Calcium imaging was performed before, during, and after the electrical stimulation to track dynamic changes in intracellular Ca^2+^ concentration [Ca^2+^]_i_ over time. Representative calcium imaging traces reported in Figure 8b–d indicate that, in response to the electrical stimulus, astrocytes on laser‐scribed electrodes exhibit an oscillatory Ca^2+^ signal [24]. This behavior resembles the previously described “P‐type” response; however, in this case, the oscillation amplitude progressively decreases over time. Notably, the effectiveness of the stimulation, the magnitude and number of oscillations of the evoked astrocyte Ca^2+^ response change depending on the electrical resistance of the electrode used to deliver the electrical stimulus (from *R*_1_ to *R*_3_, Figure 8b–d). Specifically, astrocytes stimulated with electrodes produced at higher laser fluence (0.72 J cm^−2^)—resulting in lower electrical sheet resistance—exhibit strong Ca^2+^ oscillations (*R*_1_, Figure 8b). In contrast, stimulation with electrodes fabricated at lower laser fluence—resulting in higher electrical sheet resistance—leads to a reduced Ca^2+^ signal (*R*_2_, Figure 8c). Interestingly, stimulation with electrodes displaying the highest electrical sheet resistance causes an almost complete abolition of the Ca^2+^ response (*R*_3_, Figure 8d).

**FIGURE 8:**
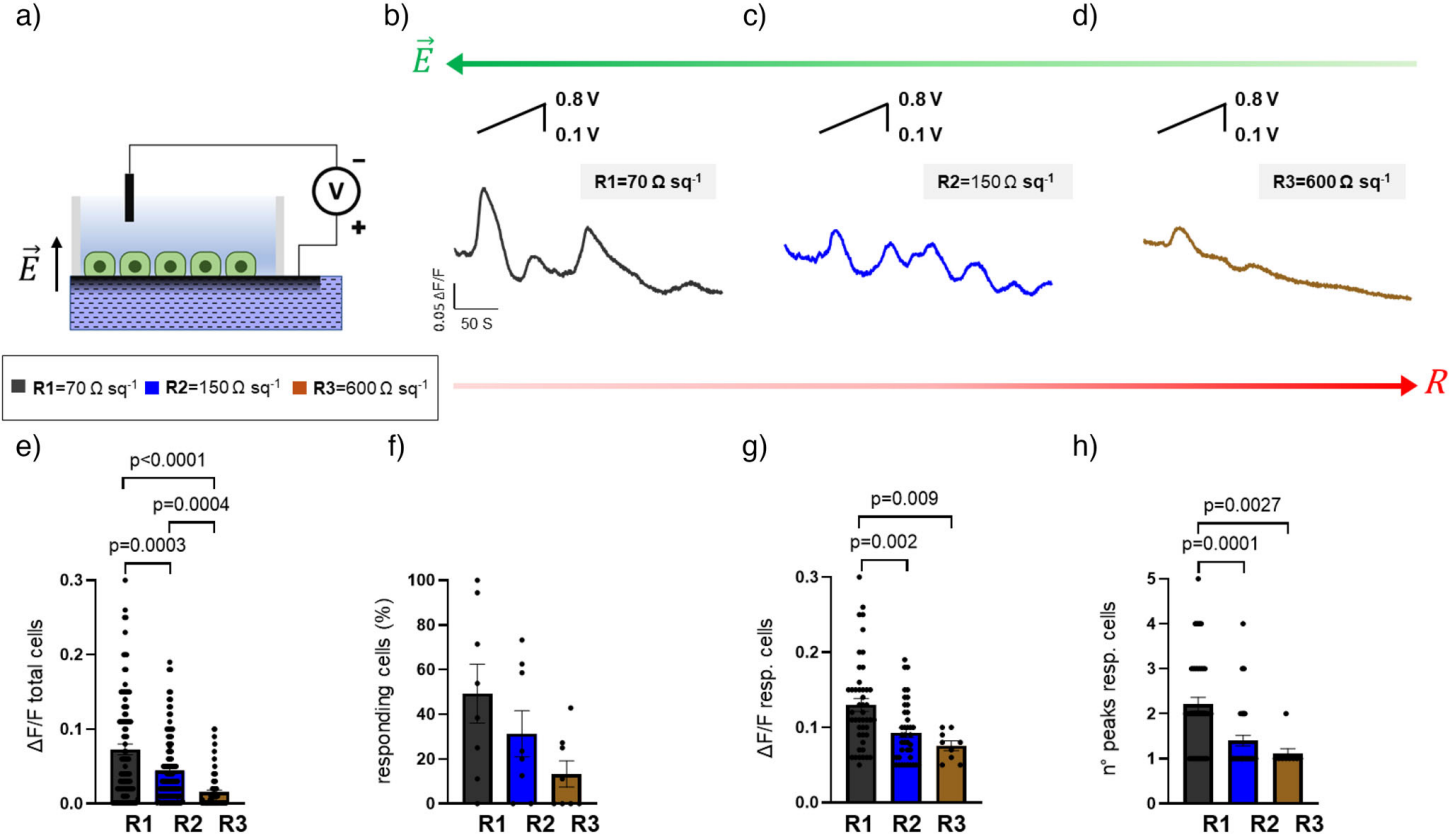
(a) Side view of a schematic representation of the experimental system. Pristine PLA‐GO composite is shown in blue, laser‐treated PLA‐GO in black. (b–d) Typical [Ca^2+^]_i_ variations observed in astrocytes grown on each laser‐scribed PLA/GO‐0.5% electrode, respectively *R*_1_ *, R*_2_ *, R*_3_, in an external standard solution containing Ca^2+^, when stimulated according to the protocol described. (e–h) Bar–dot graphs of averaged maximal fluorescence variation measured in the total number of cells (e), percentage number of responding cells (f), maximal fluorescence variation measured in responding cells (g), and number of peaks measured in responding cells (h). For *R*_1_, ntot = 98, nresp = 47, *d* = 8, *N* = 4; for *R*_2_, ntot = 107, nresp = 40, *d* = 8, *N* = 4; for *R*_3_, ntot = 85, nresp = 9, *d* = 8, *N* = 4 (ntot = total number of cells, nresp = number of responding cells, *d* = number of devices tested, *N* = number of experiments). Data are presented as mean ± s.e.m. Statistical significance was calculated via one‐way ANOVA with Bonferroni post‐test.

Statistical analysis confirms that electrical stimulation of astrocytes using laser‐scribed electrodes with progressively increasing electrical resistance results in a significant reduction in the maximal average fluorescence variation, reflecting a decrease in intracellular Ca^2+^ levels, across the entire cell population (Figure 8e). Moreover, the percentage of responding cells (Figure 8f) and the characteristics of their Ca^2+^ signaling, including the maximal averaged fluorescence variation (Figure 8g) and the number of Ca^2+^ peaks (Figure 8h), gradually decrease as the electrical resistance of the electrodes increases.

Collectively, these results suggest a correlation between the electrical resistance of the laser‐treated PLA/GO electrodes and both the amplitude and temporal dynamics of astrocytic Ca^2+^ signaling. Importantly, the ability to precisely tune the electrical sheet resistance of laser‐scribed electrodes to control the strength of astrocyte Ca^2+^ responses highlights laser‐treated PLA/GO electrode as a promising key tool to effectively address the glia‐targeted neural stimulation [55].

We reported in our previous work [24] that a uniform, highly conductive layer of rGO on ITO creates a resistor‐like system, with the astrocyte cells floating at the same potential as the electrode; electrical stimulation causes an electric field *E* opposite to the potential of the upper membrane cell, hindering the intake of external Ca^2+^, causing the release of Ca^2+^ stored in endoplasmic reticulum or in mitochondria. This release mechanism can be observed in the form of fast oscillation peaks of Ca^2+^ lasting on a scale of seconds (“P‐type” behavior). The laser‐treated PLA/GO electrodes, although chemically and morphologically different from rGO on ITO electrodes, show short, fast peaks in [Ca^2^^+^], each lasting less than 50 s (Figure 8b,c), when the electrodes are sufficiently conductive. This behavior is analogous to the previously mentioned “P‐type” response.

To investigate the underlying mechanisms of the observed Ca^2+^ oscillations, pharmacological methods were employed (Figure 9). Replacing the standard saline solution (CTRL) with a Ca^2+^ ‐free external solution (0 EXT Ca) did not significantly affect the amplitude of the Ca^2+^ signals or the number of peaks in responsive cells after the electrical stimulation delivered with *R*_1_, *R*_2_, *R*_3_ (Figure 9d,e). However, although not statistically significant, the proportion of responding cells decreased when astrocytes were stimulated with the lower sheet resistance electrode *R*_1_ (Figure 9f), suggesting that extracellular Ca^2+^ influx may play a role in generating the observed response.

**FIGURE 9:**
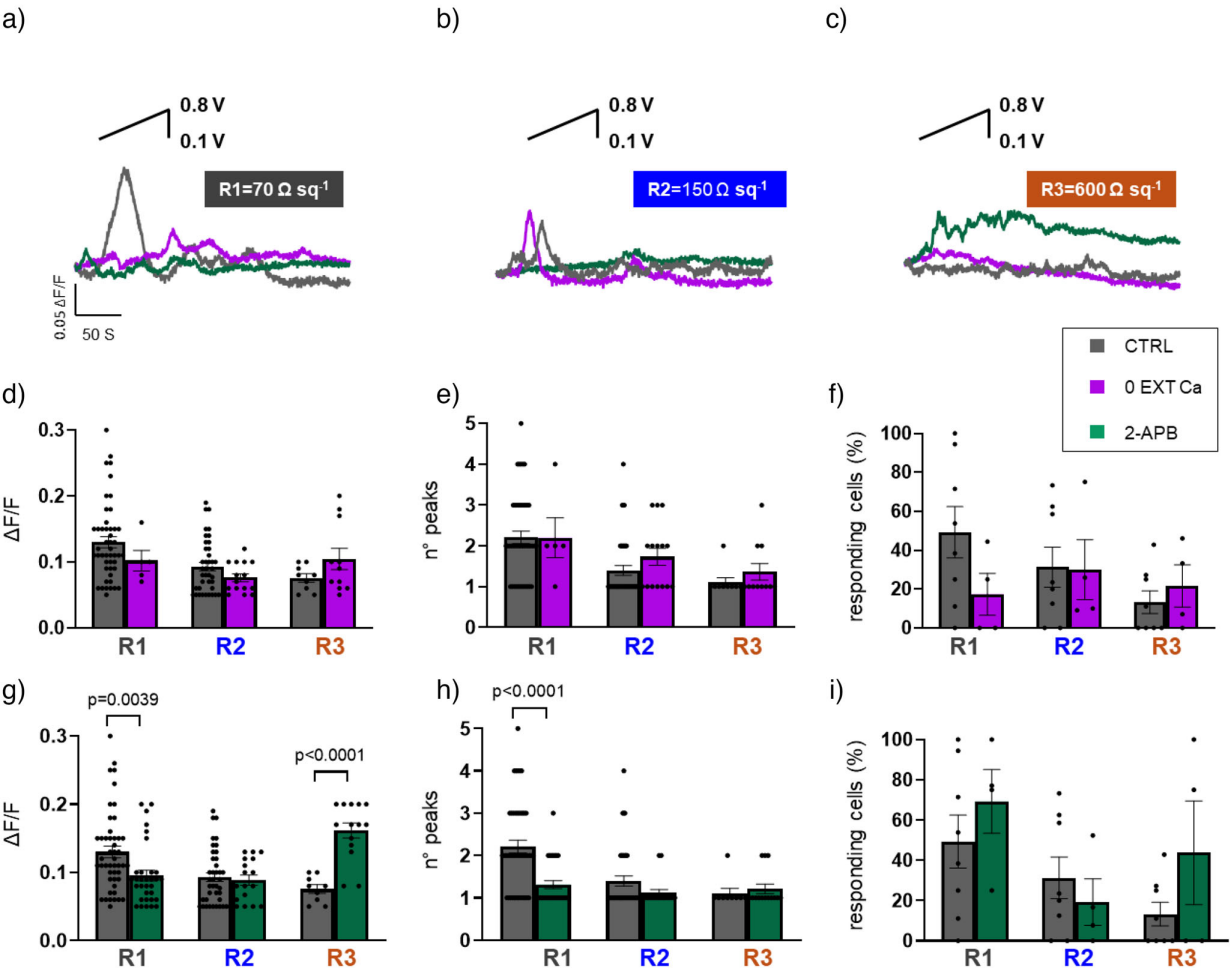
Pharmacological characterization of astrocyte Ca^2+^ response to the electrical stimulation with the PLA/GO platform. (a–c) Typical [Ca^2+^]i variations observed in astrocytes stimulated with each laser‐scribed PLA/GO‐0.5% electrode, respectively *R*_1_, *R*_2_, *R*_3_, in presence of calcium‐free extracellular solution (0 EXT Ca) and of IP3 antagonist 2‐aminoethoxydiphenyl borate (2‐APB), compared with standard saline solution (CTRL). (d–i) Bar–dot graphs of averaged maximal fluorescence variation measured in responding cells (d and g), number of peaks measured in responding cells (e and h), percentage of responding cells (f and i), in presence of 0 EXT Ca (d–f) and 2‐APB (g–i). CTRL condition: for *R*_1_, nresp = 47, *d* = 8, *N* = 4; for *R*_2_, nresp = 40, *d* = 8, *N* = 4; for *R*_3_, nresp = 9, *d* = 8, *N* = 4. 0 EXT Ca condition: for *R*_1_, *n* *resp* = 5, *d* = 4, *N* = 4; for *R*_2_, nresp = 15, *d* = 4, *N* = 4; for *R*_3_, nresp = 11, *d* = 4, *N* = 4 (nresp = number of responding cells, *d* = number of devices tested, *N* = number of experiments). 2‐APB condition: for *R*_1_, *n* *resp* = 32, *d* = 4, *N* = 4; for *R*_2_, nresp = 17, *d* = 4, *N* = 4; for *R*_3_, nresp = 14, *d* = 4, *N* = 4 (nresp = number of responding cells, *d* = number of devices tested, *N* = number of experiments). Data are presented as mean ± s.e.m. Statistical significance was calculated via one‐way ANOVA with Bonferroni post‐test.

Next, we examined whether blocking intracellular Ca^2+^ release pathways would alter the observed oscillations, thereby clarifying the contribution of internal Ca^2+^ stores to the response (Figure 9g–i). Application of 2‐aminoethoxydiphenyl borate (2‐APB, 100 µM), an antagonist of inositol 1,4,5‐trisphosphate (IP3) receptors, resulted in a significant reduction in Ca^2+^ response amplitude following *R*_1_‐mediated electrical stimulation compared to stimulation in standard saline. This effect was also accompanied by a decrease in the number of Ca^2+^ peaks, suggesting a possible contribution of IP3 to the Ca^2+^ response. In contrast, no significant effect was observed under *R*_2_ stimulation. Interestingly, when astrocytes were stimulated using the highest sheet resistance electrode (*R*_3_) in the presence of 2‐APB, the Ca^2+^ signal amplitude was significantly increased, along with slower signaling dynamics. This may indicate that, when intracellular Ca ^2+^ stores are inhibited, alternative mechanisms such as extracellular Ca^2+^ influx may become more prominent. Future pharmacological studies could provide a deeper insight into this hypothesis.

We have previously shown that changing the laser fluence affects, along with the electrode electrical resistance, also other interdependent properties, such as surface morphology and chemical composition. We hypothesize that these variations collectively modulate the magnitude of the electric field and the induced ion movement at the cell‐electrolyte interface, thereby influencing intracellular signaling pathways responsible for Ca^2^^+^ release from internal stores and progressively tuning the intensity of the “P‐type” response down to its near‐complete suppression. Therefore, while our results highlight a clear correlation between electrode sheet resistance and astrocytic Ca^2^^+^ responses, the complexity of the system suggests that the observed biological effects cannot be exclusively attributed to variations in electrical resistance alone, and that also the combined effects of the additional electrode characteristics, such as capacity, effective volume and surface area, and chemical composition, may play an important role. Further investigations will be required to decouple these contributions and specifically address each parameter.

Noteworthy, at difference with previous results, this behavior can be observed on a versatile material widely available and processable like PLA and it can be tuned across different regions of the platform by applying specific laser fluences, enabling locally differentiated astrocyte stimulation.

These results confer the laser‐scribed PLA/GO‐0.5% device with an innovative signal‐switch on capability, which may be particularly advantageous when astrocyte activity is weakened or impaired under pathological conditions. These findings open future perspectives for the application of laser‐scribed PLA/GO platform in the development of selective, flexible and tunable bioelectronic interfaces and targeted therapeutic strategies, in the study and treatment of the brain. Electrochemical sensors can be combined to the electrical stimulation to achieve, at the same time, direct control of the effect imparted to the glial cells.

## 3 Conclusions

PLA/GO platforms are proposed here as biocompatible and biodegradable devices, becoming conductive after treatment with a CO_2_ laser. The fluence used for the realization of the traces not only affects the sheet resistance, but also the chemical structure and morphology of the electrode platform, finally resulting in electrochemical sensors that take advantage of the suitable *R_CT_* and electrocatalytic properties. PLA/GO platforms were successfully applied to the electrochemical detection of all the molecules involved in the Adr biosynthetic process, opening to the possibility of studying the generation of Adr at cellular levels.

Furthermore, their biocompatibility and ability to modulate astrocytic calcium signaling through controlled electrical stimulation highlight their potential as innovative interfaces for neuroscience research and bioelectronic medicine. Overall, the proposed approach offers a scalable and cost‐effective route to disposable, multifunctional devices that integrate sensing and stimulation capabilities, paving the way for sustainable solutions in health monitoring and neurotechnology.

Accumulating evidence supports a dynamic, bidirectional regulation of neuronal communication by astrocytes, which can both detect and integrate the neuronal signals through neurotransmitter receptors and regulate synaptic neurotransmission via gliotransmitters release. Interestingly, recent studies show that the neurotransmitter noradrenaline acts on astrocytes β2‑adrenergic receptors to regulate sleep homeostasis and metabolic clearance [56]. In this context, the tunable electrochemical performance of the PLA/GO platform, combined with its biocompatibility and selective stimulation capability, makes it potentially suitable for evoking selective dynamics and probing key molecules, such as noradrenaline, in brain astrocytes.

Notably, the recent recognition of active signaling pathways between astrocytes and neurons in a process known as “gliotransmission” has fundamentally shaped the traditional view of brain function. However, questions remain regarding how astrocytes influence synapses, neuronal circuits and ultimately the behavior. In this regard, extending our PLA/GO platform‐based approach to the detection of key neuro/gliotransmitters following the application of different electrical stimulation protocols in astrocytes could provide a deeper insight into neuron‐glia communication and gliotransmission [21, 22].

The ability of the PLA/GO platform to selectively stimulate astrocyte dynamics, while simultaneously allowing the sensitive monitoring of the neurochemical environment, could inspire the future development of advanced interfaces and devices for precise stimulation and sensing of astrocytes. Such platforms would serve as versatile tools for investigating fundamental mechanisms underlying brain function, with potential applications in the therapy of severe neurological diseases [57].

## 4 Materials and Methods

### 4.1 Preparation of PLA/GO Films

Ingeo PLA 4060D (PLA), consisting of an amorphous polymer with an L‐lactide content of around 88 wt%, was kindly provided by NatureWorks LLC (Minnetonka, MN). The preparation of PLA dispersions was adapted from a previous publication, where the synthesis of the mPEG‐PLA‐mPEG block copolymers, acting as a surfactant, is also reported [16]. 0.780 g of surfactant was dissolved into 26 mL of ultrapure water at room temperature and 3.740 g of PLA was dissolved in 34 mL of ethyl acetate at 50°C. The organic phase was added to the aqueous phase in a 100 mL glass beaker at room temperature and homogenized at 0°C using a Hielscher UP200St ultrasonicator (Hielscher Ultrasonics, Teltow, Germany) equipped with a 14 mm probe, set at 50% intensity for 30 s and then at 80% intensity for 90 s. The water/oil emulsion was mechanically stirred under a fume hood until the removal of the organic solvent was complete. The resulting water dispersion of PLA nanoparticles exhibited particle sizes in the range of 340–420 nm as measured by DLS.

4 mg/mL GO solution (lateral size: D90 5–7 µm, D50 2–4 µm, D10 1–2 µm, monolayer content >95%, carbon content: 49%−56% Oxygen content: 41%–50%, from product datasheet) was supplied by Graphenea. Due to the presence of oxygen‐containing functional groups such as hydroxyl, carboxyl, carbonyl, and epoxy on its basal plane and edges, GO can be dissolved in water and other solvents, and can be easily processed to produce films, coatings or composites. To improve its conductivity, graphene oxide is often chemically or thermally reduced to form reduced graphene oxide. This process removes many oxygen groups and partially restores the percolation pathways of conductive aromatic carbon [58].

PLA/GO dispersions were obtained by adding dropwise the GO solution to the PLA dispersion under stirring. Three different quantities of GO (0.5%, 1%, or 2% relative to the solid content of PLA + PEG‐PLA‐PEG) were tested, forming the PLA/GO‐0.5%, PLA/GO‐1%, and PLA/GO‐2% composites, respectively. We also prepared a further material without any addition of GO (PLA/GO‐0%) as a reference to assess the influence of GO in the properties of the composite materials. Self‐standing films of PLA and PLA/GO were prepared via solvent casting, after pouring the solutions onto polystyrene capsules with a diameter of 5.5 cm and leaving them to dry at 60°C in an oven. The weight of the films prepared was around 500 mg.

GO‐100% films were prepared by solvent casting 5 mL of a 4 mg/mL GO aqueous solution (Graphenea) into polystyrene dishes (5.5 cm diameter) and allowing them to dry at room temperature.

Preparation of additional reference materials is reported in Supporting Information.

### 4.2 Laser Treatments

Laser scribing was performed using a Trotec Speedy 360 flexx machine featuring a CO_2_ laser source with *λ* = 10.6 µm. The waist radius *r* is 97 µm. The laser operates in Pulsed Width Modulation (PWM type) with an optical power output of up to 80 W. Laser‐scribed tracks were performed by drawing lines of 1 cm of length separated by a distance *d* to form rectangles 3 mm × 10 mm. The laser‐patterned rectangles were produced by testing different laser parameters and analyzing how these affect the electrical properties of the material by measuring their respective sheet resistance.

Average laser power (*P*), laser fluence (*F*), scanning speed (*S*), and line spacing (*d*) were tested in the range of 4.8–7.2 W, 0.54–0.81 J cm^−2^, 43−85 mm s^−1^, and 25–200 µm, respectively. The frequency of the pulses used in all the laser treatments was 60 kHz. Laser fluence, which is the energy content of the pulse falling on the substrate per unit area, was calculated by the following equation and used as a key parameter for laser scribing modification:

$$F=\frac{2\times P}{\mathrm{Frequency}\times \pi r^{2}}$$

### 4.3 Characterization Techniques

The biodegradation tests were carried out in cylindrical glass containers of 500 mL capacity in which we added a layer of 5 g mature compost (ACFA compost from Enomondo, Faenza, Italy) mixed with 10 g perlite. This layer was surrounded by two layers of 15 g perlite below and above it. The humidity content of compost and perlite was adjusted and maintained at 50%. The role of perlite is to ensure gas exchange while compost serves as a microbial inoculum. The test materials were placed in the core of the compost layer at a ratio of 40 mg material/g of compost (200 mg of material). The containers were incubated at 58°C in the dark and the tests were carried out in triplicate. The CO_2_ evolved during biodegradation was trapped by means of 20 mL of 0.5 M NaOH solution contained in a beaker set in each vessel. Every 2–7 days the beakers were retrieved and replaced with the same amount of fresh NaOH solution. The retrieved NaOH solutions were titrated with 0.5 M HCl and the amount of adsorbed CO_2_ was determined. The percentage biodegradation was calculated by the ratio of the CO_2_ produced from the test material to the maximum theoretical amount of CO_2_ that can be produced from the test material. The maximum theoretical amount of CO_2_ produced was calculated from the measured total organic carbon (TOC) content [59]. The test comparing different GO contents in PLA and the one comparing laser‐treated and untreated PLA/GO‐2% were carried out at different times but using the same compost source. Laser‐treated PLA/GO‐2% composites were patterned on the surface at *F* = 0.77 J cm^−2^, *S* = 43 mm s^−1^, *d* = 25 µm.

DSC measurements were acquired on a DSC 8000 instrument equipped with an IntraCooler II cooling device (PerkinElmer Inc., Shelton, USA). The following temperature program was followed: heating from 25°C to 200°C, 5 min isotherm, cooling down to −70°C, 5 min isotherm, heating up to 200°C. The heating and cooling were carried out at 10°C min^−1^.

TGA was acquired on a TGA 4000 (PerkinElmer Inc.) instrument and data analysis was carried out on Pyris software. 5–10 mg of each sample were heated at 10°C/min in an alumina pan from 30°C to 900°C under a 30 mL/min nitrogen flow.

For sheet resistance measurements, 3 mm × 10 mm structures were fabricated and connected at the borders with silver paint. The electrical resistance was measured by a two‐probe method using a Keithley 2450 Source Meter. Sheet resistance (*R_S_*) is correlated to measured resistance by the general formula: *R_S_* *= R W L^−1^*, where *R* is the resistance across the track; *W* and *L* are the track width and length, respectively. Sheet resistance values were reported as the arithmetic mean ± standard deviation, calculated from measurements performed on multiple tracks.

The investigation of the thickness of the laser‐scribed conductive track was performed by a digital microscope (Olympus BX53 M) and a SEM (ZEISS LEO 1530 FEG), that were used to observe both the surfaces and the cross sections of the modified areas. For SEM pictures the energy of electrons was 5 keV and the signal was acquired using an in‐lens detector at a working distance of 3−5 mm.

Chemical analysis of the structure was performed with a Raman spectrometer (microRaman Renishaw) using a HeNe laser with a wavelength of 632 nm. Each spectrum is the result of 10 accumulations of 100 s.

EDX maps were acquired at 10 keV with Bruker Quantax System applied to SEM Zeiss EVO LS10 to evaluate the change in composition of the laser‐scribed part compared to the pristine.

XRD measurements were performed on a Rigaku SmartLab diffractometer equipped with a rotating‐anode Cu source. Two geometries were used: (i) (specular *θ*–2*θ*) scans to probe the entire film thickness and (ii) grazing incidence out‐of‐plane scans (incidence angle 0.2°) to selectively probe the laser treated surface layer. For the measurements, the samples were mounted on glass slides and secured with adhesive tape.

### 4.4 Electrochemical Tests

Electrochemical tests were performed using an Autolab PGSTAT128N (Metrohm). The area of the working electrode (0.09 cm^2^) was defined using a hydrophobic and insulating nail on conductive laser tracks (*F *= 0.54–0.81 J cm^−2^, *S *= 43 mm s^−1^, *d *= 25 µm), whereas the connection with the potentiostat was established using a silver ink track and a filament of Cu. For preliminary tests, the 3‐electrode cell was completed by including an external Pt wire and an Ag/AgCl/KCl (3 M) as the counter and the reference electrode, respectively. Further tests for the electrochemical detection of Adr were performed with a 3‐electrode cell entirely obtained on PLA/GO‐2%, by depositing a small amount of Ag paste to fix the potential of the pseudo‐reference electrode. The stability of the pseudo‐reference electrode was checked by monitoring the open‐circuit potential (OCP) in 0.1 M KCl both in water and in artificial sweat solutions for 1800 s (see Figure S12). The analyses were carried out at room temperature and in equilibrium with the atmosphere, that is, in the presence of O_2_. 100 µL of the test solution was dropped on the PLA/GO surface to perform the analysis.

Commercial CSPE were used as a reference to compare the electrochemical properties of laser‐scribed PLA/GO‐2% platforms. The potential of the pseudo‐reference electrode was fixed in this case by adding 0.1 M KCl to all solutions tested. All the potentials herein reported are finally corrected to an Ag/AgCl/3 M KCl reference, using the 0.5 mM Fc(OH)_2_, 0.1 M KCl solution as an internal standard, to allow the direct comparison with the responses recorded at PLA/GO surfaces.

The electrochemical behavior of laser‐scribed PLA/GO surfaces was first studied by cyclic voltammetry (CV), recording voltammetric responses at different scan rates in a 0.5 mM Fc(OH)_2_, 0.1 M KCl solution. Further tests to evaluate the resistance to charge transfer (*R_CT_*) were carried out using EIS by recording the Nyquist plots (i.e., Z″ vs. Z′) in a 1:1, 5 mM [Fe(CN)_6_]^3‐/4−^, 0.1 M KCl solution. These measurements were performed at OCP, in the 50 mHz–10 kHz frequency range, using an amplitude of 0.01 V and recording 50 frequencies per decade. In all cases, the Nyquist plots were fitted with a Randles circuit, and the high‐frequency region evidenced the typical semicircle, whose diameter gave us a measure of the *R_CT_* of the material.

The electrocatalytic performance of laser‐scribed PLA/GO‐2% was evaluated toward the oxidation of several organic species present in biological fluids, namely adrenaline (Adr), ascorbic acid, NADH, uric acid, tyrosine (Tyr), L‐Dopa, dopamine (Dop), and noradrenaline (Nor) dissolved in artificial sweat. The composition of this last solution, resembling the human fluid, was 25.5 g L^−1^ KCl, 17.5 g L^−1^ NH_4_Cl, 5 g L^−1^ urea, 2.5 g L^−1^ CH_3_COOH, finally setting the pH to 4.7. CV traces recorded at a 0.02 V s^−1^ scan rate were used to preliminary test the electrocatalytic properties of the electrode platform, whereas DPV was then used to better assess the applicability of the device in electrochemical sensing. These measurements in this last case were performed by applying a modulation amplitude of 0.01 V, a modulation time of 0.1 s, a step potential of 6 mV and 0.020 V s^−1^ as the scan rate.

All electroactive species tested were provided by Sigma‐Aldrich and dissolved in ultra‐pure water (18 MΩ cm), obtained from a Milli‐Q system.

### 4.5 Astrocytes Stimulation Platform

#### 4.5.1 Electrodes Platform Preparation

Laser‐scribed PLA/GO‐0.5% samples were used for astrocytes growth and stimulation attached on glass slides by thin PDMS sheet. For each sample 3 electrodes were prepared by laser with same geometry (rectangles 2 × 20 mm separated by 3 mm of gap) at *S* = 57 mm s^−1^, *d* = 25 µm and with three different laser fluences (*F*_1_ *, F*_2_ *, F*_3_) to obtain different electrical sheet resistances: *R*_1_ = 70 ± 50 Ω sq^−1^ at *F*_1_ = 0.72 J cm^−2^, *R_2_* = 150 ± 90 Ω sq^−1^ at *F*_2_ = 0.68 J cm^−2^ and *R*_3_ = 600 ± 400 Ω sq^−1^ at *F*_3_ = 0.63 J cm^−2^. Average sheet resistance values are calculated from 6 tracks (6 samples). A plastic ring was attached to the sample to contain the cell culture bath by Sylgard184. Silver paint was used to wire the borders of the electrodes, while an insulating layer of varnish was applied to prevent mechanical damage to the electrodes during ring attachment.

#### 4.5.2 Cell Culture Preparations

Primary rat cortical astroglial cultures were prepared as described previously [60], in accordance with the Italian law on protection of laboratory animals, with the approval of bioethical committees of the University of Bologna and of the Ministry of Health (ID 1268, code number 2DBFE.N.HTT), under the supervision of the veterinary commission for animal welfare at the University of Bologna. Every effort was made to minimize the number of animals used and their suffering. Briefly, cerebral cortices were isolated from postnatal day 0–2 (*P*_0_–*P*_2_) Sprague–Dawley rat pups following removal of the meninges. The tissue was mechanically dissociated and placed in cell culture flasks containing Dulbecco's Modified Eagle Medium (DMEM)–GlutaMAX medium supplemented with 15% fetal bovine serum, 100 U mL^−1^ penicillin and 100 µg mL^−1^ streptomycin (all products were purchased from Gibco‐Invitrogen). Cultures were maintained at 37°C in a humidified atmosphere with 5% CO_2_ for 3 to 4 weeks, with medium replacement every three days. Before medium change, flasks were gently shaken to remove microglial cells growing on top of the astrocytic monolayer. Once confluent, astroglial cells were enzymatically detached using trypsin–EDTA, seeded at high density on the PLA/GO platform and maintained in culture medium supplemented with 10% fetal bovine serum.

#### 4.5.3 Cell Viability

Cell viability was evaluated by Fluoresceine Diacetate (FDA)/HOESCHT assay after 5 days in vitro. The FDA (Sigma) stock solution (5 mg mL^−1^) was diluted in phosphate‐buffered solution (PBS). Hoechst 33342 (1:2000) was added to the solution. Astrocytes plated on PLA/GO platform were incubated for 5 min at room temperature (22°C–24°C), washed with PBS and characterized using an OLYMPUS BX63 fluorescence microscope (Crisel Instruments, Rome, Italy), equipped with 40X or 20X objectives. Fluorescence images were acquired using specific filter sets to image Hoechst 33342 (Excitation: 390/22, Emission: 440) and FDA (Excitation: 475/28 nm, Emission: 525 nm). The percentage of viable/total number of cells was calculated as the percentage of cells expressing FDA (green) out of the total number of nuclei, marked with Hoechst 33342 (blue).

#### 4.5.4 Electrical Stimulation and Calcium Microfluorometry In Vitro

For experiments in primary cell culture, variations in [Ca^2+^]_i_ were monitored with calcium microfluorometry using the single‐wavelength fluorescent Ca^2+^ indicator Fluo‐4 AM (Life Technologies). Before measurements, high‐density astrocytes seeded on PLA/GO devices were loaded with 2 µM Fluo‐4 AM dissolved in standard bath solution for 40 min at room temperature. Samples were rinsed with standard bath solution after incubation. Electrical stimulation was performed by immersing the samples and an Ag/AgCl/3 M reference electrode in saline bath solution and applying voltage using a custom‐made 2612A Dual‐channel System SourceMeter instrument (Keithley). The applied voltage protocol was low enough to provide an electrical field suitable for cell stimulation, while avoiding generation of detrimental faradaic currents [61]. The voltage protocol consisted of a continuous voltage ramp increasing from 0.1 to 0.8 V in 85 s at a rate of 8.24 mV s^−1^. The total length of the experiment was 300 s, and the voltage stimulus was applied 25 s after the start of the recording. Measurements of [Ca^2+^]_i_ were performed using a fluorescence microscope (OLYMPUS BX63), equipped with a 40X objective. Fluorescence was excited using SPECTRA III light engine (Lumencor) at 475/28 nm and collected using a FITC/GFP filter at 525 nm. Camera exposure time was set to 150 ms, with an image sampling rate of 2 Hz. Data acquisition was controlled using MetaMorph software (Molecular Devices). In calcium imaging experiments, the ratio of the fluorescence intensity at each time point and the initial fluorescence was continuously recorded during the experiment (Δ*F*/*F*). Cells were considered responding to the stimulus when the maximal variation in fluorescence after the stimulus was higher than 0.05 Δ*F*/*F*. To evaluate the temporal features of [Ca^2+^]_i_ dynamics, the average number of peaks was extracted by detecting the number of fluorescence oscillations recorded over time, from the beginning of electrical stimulation until the end of the experiment.

#### 4.5.5 Solutions and Chemicals

Salts and other chemicals of the highest purity grade were purchased from Sigma. For calcium microfluorometry experiments, the standard bath solution was composed of (mM) 140 NaCl, 4 KCl, 2 MgCl_2_, 2 CaCl_2_, 10 HEPES, 5 glucose, pH 7.4 with NaOH and osmolarity adjusted to ∼318 mOsm with mannitol. Ca^2+^ ‐free extracellular solution was composed of (mM) 140 NaCl, 4 KCl, 4 MgCl2, 10 HEPES, 0.5 EGTA, pH 7.4 with NaOH and osmolarity adjusted to ∼318 mOsm with mannitol. Stock solution of 2‐aminoethoxy diphenyl borate (2‐APB, 100 mM) was prepared by dissolution in methanol and stored at −20°C.

### 4.6 Statistical Analysis

For in vitro calcium imaging experiments, somatic cellular fluorescence time series were manually extracted using Fiji ImageJ and the statistical analysis of extracted data was performed using Prism GraphPad 8.0.2. For viability experiments, fluorescence images were analyzed using Fiji ImageJ and the statistical analysis was performed using Prism GraphPad 8.0.2.

Data were compared using one way ANOVA with multiple comparisons with Bonferroni post‐test. A statistically significant difference was reported if *p* ≤ 0.05. All data are presented as mean ± s.e.m. The data were analyzed from 3 independent experiments. All the calculated means, s.e.m., *p* values, numbers of experiments (*N*) and numbers of cells or replicates (*n*) are reported in the graph and figure legend.

## Author Contributions

**Roberta Fabbri**: data curation, investigation, Writing – original draft, Writing – review and editing, visualization. **Matteo Calosi**: data curation, investigation, writing – original draft, writing – review and editing, visualization. **Alessandro Silvestri**: data curation, investigation, writing – original draft, writing – review and editing, visualization. **Vincenzo Palermo**: writing – review and editing, visualization, writing – original draft. **Giulia Cazzador**: data curation, investigation, writing – original draft, writing – review and editing, visualization. **Andrea Candini**: writing – original draft, writing – review and editing, visualization, supervision. **Monica Bertoldo**: data curation, investigation, supervision, writing – original draft, writing – review and editing, visualization. **Fabiola Liscio**: data curation, investigation, writing – original draft, writing – review and editing, visualization. **Franco Corticelli**: investigation, writing – review and editing, visualization. **Emanuele Treossi**: conceptualization, data curation, investigation, supervision, writing – original draft, writing – review and editing, visualization. **Chiara Zanardi**: conceptualization, data curation, investigation, supervision, writing – original draft, writing – review and editing, visualization. **Valentina Benfenati**: conceptualization, data curation, investigation, supervision, writing – original draft, writing – review and editing, visualization. **Andrea D'iorio**: data curation, investigation, writing – review and editing. **Marco Caprini**: writing – review and editing, resources. **Alessandra Scidà**: conceptualization, data curation, investigation, writing – original draft, writing – review and editing, visualization. **Aikaterini Konstantoulaki**: writing – review and editing, resources.

## Conflicts of Interest

The authors declare no conflicts of interest.

## Supporting information




**Supporting File**: adma74487‐sup‐0001‐SuppMat.docx.

## Data Availability

The data that support the findings of this study are available from the corresponding author upon reasonable request.
