# Supplementary material for: Sustainable Poly(Lactic Acid)/Graphene Oxide Bioelectronic Platform for Neurotransmitters Sensing and Tunable Stimulation of Astrocytes
Source: Adv Mater. 2026 Aug 9;38(58):e74487. doi: 10.1002/adma.74487 (PMC13674625; doi:10.1002/adma.74487)
Supplement: Supplementary file 1 [file ADMA-38-e74487-s001.docx]

**SUPPORTING INFORMATION**

**Sustainable Poly(Lactic Acid)/Graphene Oxide Bioelectronic Platform for Neurotransmitters Sensing and Tunable Stimulation of Astrocytes**

Alessandra Scidà^1#^, Giulia Cazzador^2#^, Roberta Fabbri^1#^, Matteo Calosi^3^, Alessandro Silvestri^2^, Andrea Candini^1^, Fabiola Liscio^4^, Franco Corticelli^4^, Katerina Aikaterini Konstantoulaki^1^, Andrea D'Iorio^3^, Marco Caprini^5,6^, Vincenzo Palermo^1^, Monica Bertoldo^3^, Valentina Benfenati^1^*, Chiara Zanardi^1,2^*, Emanuele Treossi^1^*

1) Institute of Organic Synthesis and Photoreactivity (ISOF) – Italian National Research Council (CNR), 40129 Bologna, Italy. 2) Department of Molecular Sciences and Nanosystems, Ca’ Foscari University of Venice, Venezia, 30172, Italy. 3)Department of Chemical, Pharmaceutical and Agricultural Sciences, University of Ferrara, I-44121 Ferrara, Italy. 4) Institute of Nanostructured Materials (ISMN) – Italian National Research Council (CNR), 40129 Bologna, Italy. 5) Department of Pharmacy and Biotechnology (FaBit), University of Bologna, Bologna, Italy. 6) IRCCS Istituto delle Scienze Neurologiche di Bologna, Bologna, Italy.

Correspondence: Valentina Benfenati (valentina.benfenati@cnr.it), Chiara Zanardi (chiara.zanardi@unive.it), Emanuele Treossi (emanuele.treossi@cnr.it)

\# These authors contributed equally.

Fig. S1: Optical pictures of PLA/GO films patterned by laser irradiation: a) PLA/GO-0% lasered at different laser fluence from 0.54 to 0.72 J cm^-2^; b) PLA/GO-1% lasered at different laser fluence from 0.59 to 0.77 J cm^-2^; c) PLA/GO-2% lasered at different laser fluence from 0.59 to 0.77 J cm^-2^. d) Average sheet resistance of laser patterned rectangles 3 × 10 mm as a function of the laser fluence for different PLA/GO concentrations, calculated from 4 tracks (2 samples). Average sheet resistance of laser patterned rectangles 3 × 10 mm for PLA/GO-2% at laser Fluence= 0.77 J cm^-2^ as a function of e) scanning speed and f) line spacing calculated from 2 tracks (1 sample). The error bars represent the standard deviation.

In Fig. S1a-c the optical pictures of PLA/GO-0%, PLA/GO-1% and PLA/GO 2% laser-treated at different fluences are reported. PLA/GO-0% shows signs of material softening at 0.54 J cm^-2^ and 0.59 J cm^-2^ while, already starting from 0.63 J cm^-2^, a discontinuous material with numerous holes is obtained. No evidence of electrically conductive trace formation has been detected in laser treated PLA/GO-0%.

The sheet resistance of the laser treated PLA/GO composites can be tuned by changing the laser parameters such as the laser fluence (Fig. S1d), the scanning speed (Fig. S1e) and the line spacing (Fig. S1f). By increasing the laser fluence at constant scanning speed (in this case S= 43 mm s^-1^), it is possible to increase the duty cycle and consequently the exposure time, which enables a decrease in sheet resistance (Fig. S1d). On the contrary, by increasing the scanning speed, at constant fluence, the exposure time decreases, and the sheet resistance increases (Fig. S1e). When laser scribing is performed by vectorial cutting, the line spacing is an important parameter to take into account, indeed when the spacing is smaller than waist diameter, as in this case, most of the lasered area is subjected to multiple laser scanning. In particular, the increase of the line spacing leads to fewer overlaps between the scanning lines and consequently to a higher sheet resistance, as shown in Fig. S1f for constant fluence (0.77 J cm^-2^) and constant scanning speed (43 mm s^-1^). Except for the test in Fig. S1f, all other laser treatments were performed with a line spacing of 25 µm.

Additional reference materials were prepared to assess whether conductive structures could be generated by laser treatment in PLA composites containing fillers other than GO. For this purpose, boron nitride (BN) and silicon dioxide nanoparticles (SiO₂NP) were selected as electrically insulating, non-carbonaceous reference fillers. BN provides a layered two-dimensional morphology, whereas SiO₂NP was selected as a non-carbonaceous inorganic filler representing a material class known to interact with CO₂ laser irradiation through thermally driven processes. The use of electrically insulating, non-carbonaceous fillers allows exclusion of contributions arising from the intrinsic electrical conductivity of the filler remaining within the laser-treated track. PLA-based composites incorporating BN and SiO₂NP were prepared by adding 4 wt% of BN (HeBoFill® 630 boron nitride powder, median particle size 9 μm, purity B + N >97%, specific surface area 30 m² g⁻¹, Henze) or 20 wt% SiO₂NP (PL-SiO10-30p100m, average particle size 10 nm, purity >99.5%, specific surface area ~320 m² g⁻¹, PlasmaChem GmbH) to the PLA dispersion under stirring. The dispersions were cast into polystyrene dishes (5.5 cm diameter) and dried at 60 °C until complete solvent evaporation, yielding self-standing PLA/BN-4% and PLA/SiO₂NP-20% films.

 Fig. S2: a) Optical pictures of laser-treated surface of different composites at the laser fluence marked on the left: PLA/GO-0%, PLA/BN-4%, PLA/SiO_2_NP-20% and GO-100%. b) Average sheet resistance of laser patterned rectangles 3 × 10 mm as a function of the laser fluence for GO-100% composite calculated from at least 3 tracks on 3 samples. The error bars represent the standard deviation.

Laser processing was performed on PLA/GO-0%, PLA/BN-4%, PLA/SiO₂NP-20%, and GO-100% samples at different fluences (0.54–0.72 J cm⁻²), using a scanning speed of 43 mm s⁻¹ and a line spacing of 25 µm to produce rectangular tracks (3 × 10 mm). The laser-treated areas are shown in the optical images reported in Figure S2a. No conductive tracks were obtained for the PLA/GO-0%, PLA/BN-4%, and PLA/SiO₂NP-20% samples. At higher fluences, the irradiated regions underwent extensive material removal, leading to the complete removal of the material and formation of through-holes having the shape of the written paths. In contrast, laser-treated GO-100% samples produced conductive tracks at all investigated fluences, exhibiting sheet resistance values as low as 22 Ω sq⁻¹. However, the resulting tracks were mechanically fragile and frequently showed cracking and partial fragmentation. Figure S2b reports the average sheet resistance values of the laser-treated GO-100% samples that retained structural integrity after processing.

Fig S3: Top: optical microscopy images of PLA/GO-2% surface laser-treated at the laser fluence marked above; bottom: corresponding SEM images of PLA/GO-2%. The scale refers to each image of the series.

Fig S4: Optical microscope images of the cross sections of PLA/GO-2% samples pristine and after laser irradiation at different laser fluences. Red arrows indicate the sample surface modified by the laser.

Fig S5: Top: SEM images of a) top surface of the transition between lasered track and pristine PLA/GO-2% material at laser fluence= 0.77 J cm^-2^; b), c), d) cross sections at different magnifications of the track lasered at fluence= 0.77 J cm^-2^ and the remaining PLA/GO substrate. Bottom: the cross section of the track lasered at a fluence of 0.81 J cm^-2^ and the remaining PLA/GO-2% film captured by SEM image e) and respective EDX maps for f) carbon and g) oxygen; h) fusion of images e) f) g).

Figure S6: (a) Specular θ–2θ scans of PLA/GO-2% films written at increasing laser fluences. (b) Integrated area of the (002) peak *vs* laser fluence. (c) Coherence domain size of (002) graphene planes *vs* laser fluence. The coherence length was estimated from the peak broadening using the Scherrer equation. Error bars in panels (b,c) represent the uncertainty obtained from peak fitting/line-shape analysis. Signal from pristine PLA/GO-0% and from the tape substrate used are also reported.

Figure S7: Biodegradation curves in composting conditions of PLA/GO-2% samples before and after laser scribing at fluence 0.77 J cm^-2^; error bars represent standard error (n = 3).

Figure S8 - CV responses recorded at different scan rates in 0.5 mM Fc(OH)_2_, 0.1M KCl solution, with PLA/GO-2% electrodes obtained at laser fluence of a) 0.59 J cm^-2^, b) 0.63 J cm^-2^, and c) 0.81 J cm^-2^ in comparison to analogous responses collected at CSPE (d).

Figure S9 - CV responses of 1 mM a) Tyr, b) L-Dopa, c) Dop, d) Nor, and e) Adr recorded at laser scribed PLA/GO-2% electrodes obtained with a fluence of 0.72 J cm^-2^ (red lines) and CSPE (blue lines). The lowest picture of each frame reports the comparison of the responses collected in the anodic region subtracted for the relevant blank signal (dotted black lines).

Figure S10 - DPV responses recorded with a) the 3-electrode platform entirely realized with laser scribed PLA/GO-2% and b) with CSPE in artificial sweat solutions containing different amounts of Adr (from 0.04 to 1 mM). The inset shows the relevant calibration plot.

Figure S11. DPV responses recorded with a) laser scribed PLA/GO-2% (0.72 J cm^-2^ laser fluence) and c) CSPE in 0.1mM Adr, 0.1M artificial sweat and 0.1 M KCl during the first (black lines) and the third (colored lines) analyses in solutions at different concentrations to define the calibration plot. The entity of surface variation due to the analysis of Adr is highlighted by EIS plots recorded in Fe(CN)_6_^2+/3+^, 0.1M KCl solution with b) laser scribed PLA/GO-2% and d) CSPE before (black lines) and after (colored lines) the analysis of the various Adr solutions in the 0.04-1 mM concentration range repeated three times. e) Graphic representation of the variation in faradic current of DPV traces collected during the first and the third analysis; error bars represent the standard deviation calculated by considering measurements with three independent sensors used under the same conditions.

Figure S12 – Open Circuit Potential (OCP) responses recorded in 0.1 M KCl a) in water and b) in artificial sweat.
